# Reversible coordination of N_2_ and H_2_ to a homoleptic *S* = 1/2 Fe(i) diphosphine complex in solution and the solid state†
†Electronic supplementary information (ESI) available: Full experimental details and characterisation data. CCDC 1451414. For ESI and crystallographic data in CIF or other electronic format see DOI: 10.1039/c8sc01841c


**DOI:** 10.1039/c8sc01841c

**Published:** 2018-07-18

**Authors:** Laurence R. Doyle, Daniel J. Scott, Peter J. Hill, Duncan A. X. Fraser, William K. Myers, Andrew J. P. White, Jennifer C. Green, Andrew E. Ashley

**Affiliations:** a Department of Chemistry , Imperial College London , London SW7 2AZ , UK . Email: a.ashley@imperial.ac.uk; b Inorganic Chemistry Laboratory , University of Oxford , Oxford OX1 3QR , UK

## Abstract

The synthesis and characterisation of the *S* = 1/2 Fe(i) complex [Fe(depe)_2_]^+^[BArF4]^–^ ([**1**]^+^[BArF4]^–^), and the facile reversible binding of N_2_ and H_2_ in both solution and the solid state to form the adducts [**1**·N_2_]^+^ and [**1**·H_2_]^+^, are reported. Coordination of N_2_ in THF is thermodynamically favourable under ambient conditions (1 atm; Δ*G*_298_ = –4.9(1) kcal mol^–1^), while heterogenous binding is more favourable for H_2_ than N_2_ by a factor of ∼300. [**1**·H_2_]^+^[BArF4]^–^ represents a rare example of a well-defined, open-shell, non-classical dihydrogen complex, as corroborated by ESR spectroscopy. The rapid exchange between N_2_ and H_2_ coordination under ambient conditions is unique for a paramagnetic Fe complex.

## Abstract 2

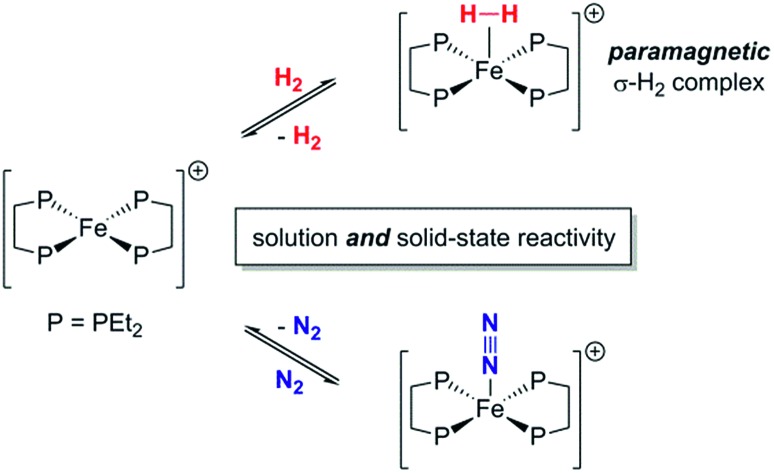
A 15 valence-electron Fe(i) species rapidly and reversibly binds N_2_ and H_2_, the latter producing a rare paramagnetic dihydrogen complex.

## Introduction

Recent years have seen great interest in the pursuit of well-defined transition metal (TM) complexes capable of catalysing the reduction of N_2_ to NH_3_ or N_2_H_4_.^1^ One of the most compelling rationales for studying such systems is that their mechanistic details can be more readily discerned, in comparison with the complex proton-coupled electron transfer (PCET) steps operative within nitrogenase enzymes.^2^ The utility of Fe in biological N_2_ fixation,^3^ and the anthropogenic Haber–Bosch process,^4^ has prompted researchers to target Fe complexes as potential synthetic catalysts. These ‘artificial’ nitrogenases employ chemical H^+^ and e^–^ sources to reduce N_2_ through PCET pathways, although this process competes with proton reduction to H_2_, which can preferentially sequester active metal sites.^5^ Facile displacement of H_2_ by N_2_ is thus an important aspect to maintaining a productive N_2_-fixing catalytic cycle, and an understanding of the binding of these small molecules to low-valent Fe centres could lead to more selective and efficient catalysts for the production of azanes. While the coordination chemistry of N_2_ and H_2_ to Fe(0) and Fe(ii) complexes is well-documented,^6^ analogous detailed studies containing Fe(i) are scant,^7^ despite the potential relevance of this low-valent oxidation state in Fe-based synthetic nitrogenases. We recently reported that the Fe(0) bisphosphine complex, Fe(depe)_2_(N_2_) [**1**·N_2_; depe = Et_2_PCH_2_CH_2_PEt_2_] is a highly selective catalyst for the PCET-mediated conversion of N_2_ to N_2_H_4_ with H^+^ and e^–^ equivalents.^8^ Herein we report the related low-spin Fe(i) species [Fe(depe)_2_]^+^ ([**1**]^+^) which is shown to reversibly coordinate N_2_ or H_2_, with the latter being a rare case of a thoroughly-characterised paramagnetic σ-H_2_ complex. Furthermore, this behavior is found to occur both in solution and in the solid-state.

## Methods

The ESR spectroscopic methods used for characterizing molecular structure are based on the following spin Hamiltonian, representing the electron Zeeman, nuclear Zeeman, hyperfine and nuclear quadrupole components, respectively.
1

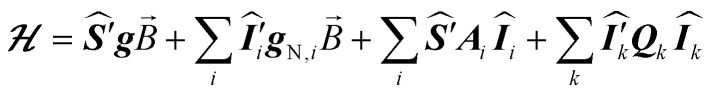




The bold symbols are 3 × 3 tensors (or matrices) and the vector of Cartesian spin operators are defined in appropriate Hilbert space eigenbasis. The third term of the spin Hamiltonian, hyperfine or 
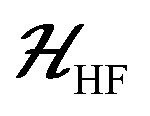
 is composed of an isotropic component, *a*_iso_, transformed to a 3 × 3 matrix by, 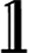 defined as the identity matrix, and an anisotropic component, ***T***.
2

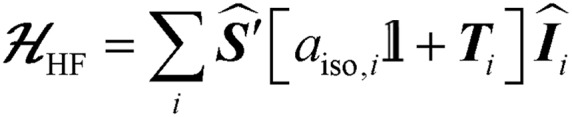




It is the anisotropic tensor component that returns the structural relations of the spin system, based on the summation of dipolar interactions between the position vectors of the central iron atom and surrounding positions, approximated as ligand nuclei position vectors.^9^
3

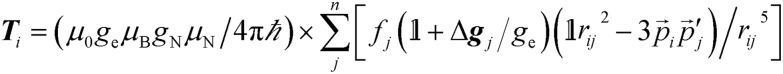




Eqn (3) includes contribution of all centres, *p*_*j*_, of spin density *f*_*j*_ at distance *r*_*ij*_ to the hyperfine interaction matrix for a single nucleus position, *p*_*i*_. The principal axis elements in the limit of an axial tensor are [–T, –T, 2T] where the perpendicular component (eqn (4)) is used to estimate a distance in a single point-dipole approximation:

4 *T* = *f*_Fe_*g*_eff_*μ*_B_*g*_N_*μ*_N_/*r*^3^

Analysis of the nuclear quadrupole term of the spin Hamiltonian of eqn (1) for an *I* = 1 nucleus (relevant to ^2^H and ^14^N in this work) can provide useful information on bonding and electronic structure,^10^ and is given by:

5 ***Q*** = *K* × diag[–(1 – *η*), –(1 + *η*), 2]

where *K* is the axial quadrupole interaction (=*e*^2^*q*_*zz*_*Q*/4*h*) and *η* is the orthorhombic asymmetry parameter. In the limit of pure quadrupole frequencies (*ν*_I_ = *A*/2) the *m*_s_ = +1/2 manifold frequencies are *ν*_+_ = *K*(3 + *η*), *ν*_–_ = *K*(3 – *η*), and *v*_0_ = 2*Kη*, for a positive hyperfine interaction.^11^ In the opposite manifold, *m*_s_ = –1/2, there are two single quantum (sq) peaks and a double quantum (dq) transition, which corresponds to *ν*_+_ in the *m*_s_ = +1/2 manifold. A formula for the double quantum frequency in the limit of small hyperfine anisotropy is:^12^

6 *ν*_dq_ = 2[*ν*_ef±_^2^ + *K*^2^(3 + *η*^2^)]^1/2^

where *ν*_ef_ is the effective frequency of the Larmor and hyperfine, *ν*_ef±_ = *ν*_I∓_*A*/2, based on the DC field and unpaired electron.

## Results and discussion

**Fig. 1:**
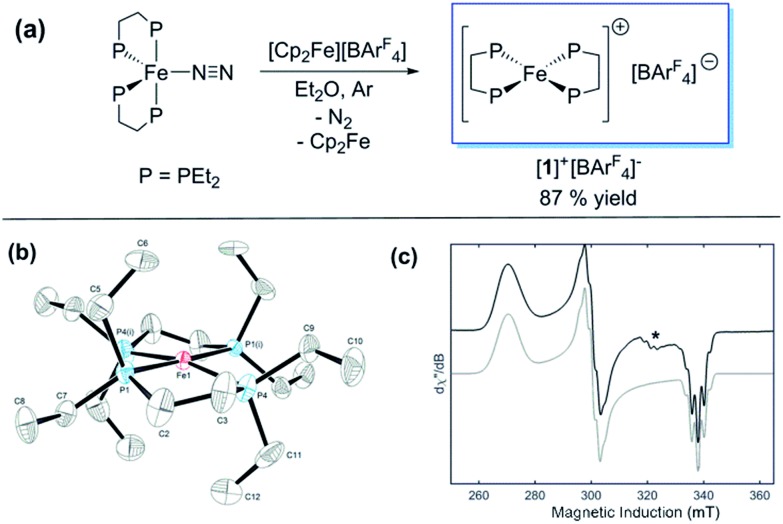
(a) Synthesis of [**1**]^+^[BArF4]^–^. (b) ORTEP diagram of the [Fe(depe)_2_]^+^ fragment in [**1**]^+^[BArF4]^–^; thermal ellipsoids shown at 30% probability, H atoms and [BArF4]^–^ counterion omitted for clarity (C and P occupancies are 0.5 as a consequence of a second symmetry-generated orientation of the two depe ligands; this has also been omitted for clarity). (c) CW X-band ESR spectrum of [**1**]^+^[BArF4]^–^ (PhMe : DFB 7 : 1; Ar; 40 K); black line: experiment; grey line: simulation. *[**1**·N_2_]^+^[BArF4]^–^ impurity due to trace N_2_ in the Ar atmosphere.

Previously, we showed that N_2_H_4_/NH_3_-producing reactions of **1**·N_2_ with the acid [Ph_2_NH_2_]^+^[TfO]^–^ (TfO = CF_3_SO_3_) formed an Fe(i) species, which was shown by X-ray crystallography to be [Fe(depe)_2_(η^1^-N_2_)]^+^[TfO]^–^ ([**1**·N_2_]^+^[TfO]^–^).^8^ Variable-temperature ESR spectra of this compound were complicated, however, which we postulated may be due to competitive N_2_*vs.* [TfO]^–^ coordination. In order to suppress the latter, and hence better resolve the behaviour of the [**1**·N_2_]^+^ fragment, we subsequently utilised the more weakly coordinating [BArF4]^–^ (BArF4 = B[3,5-{CF_3_}_2_C_6_H_3_]_4_). Thus, oxidation of **1**·N_2_ with 1 eq. [Cp_2_Fe]^+^[BAr^F^_4_]^–^ (Cp = C_5_H_5_) produced a deep-blue solution, from which an intensely blue crystalline solid was obtained following work-up and recrystallisation from Et_2_O/pentane under Ar.^13^ A single crystal suitable for X-ray diffraction was subsequently isolated which solved and refined as the 15 valence electron (VE), N_2_-free compound [Fe(depe)_2_]^+^[BArF4]^–^ ([**1**]^+^[BArF4]^–^, Fig. 1(a) and (b)). In the structure the [**1**]^+^ and [BArF4]^–^ ions are well separated, with no close C or F contacts between the anion and the Fe centre.

The [**1**]^+^ cation was found to be disordered and, while the structural model is in good agreement with the X-ray crystallographic data (with the molecular connectivity and absence of an N_2_ ligand being conclusive), the interatomic distances are approximate and will not be discussed in detail.^14^ The FeP_4_ unit is pseudo square planar and exhibits a tetrahedral distortion, as seen by a dihedral angle of 12.41(11)° between the two Fe(PP) [Fe(1)P(1)P(4) and Fe(1)P(1*i*)P(4*i*)] coordination planes, which is very similar to that observed in the approximately square-based pyramidal [**1**·N_2_]^+^[TfO]^–^ (15.39(9)°). Evidently, coordination of N_2_ results in minimal reorganisation of the [Fe(depe)_2_]^+^ fragment. While two CH_3_ groups from ligand ethyl groups from each depe moiety are directed towards the vacant axial coordination sites of the Fe centre, large Fe···H separations suggest an absence of any agostic or anagostic interactions, which is supported by DFT results (*vide infra*).^15^

Neither solid samples nor solutions of [**1**]^+^[BArF4]^–^ under Ar showed bands attributable to an *ν*_NN_ stretch in their IR or Raman spectra, further confirming the absence of N_2_ in the complex. [**1**]^+^[BArF4]^–^ is insoluble in alkanes, PhH and PhMe, yet highly soluble in THF and in the highly polar, non-coordinating 1,2-difluorobenzene (DFB). ^31^P NMR spectra (DFB, Ar) of [**1**]^+^[BArF4]^–^ are silent, whereas very broad paramagnetically-shifted resonances for the [**1**]^+^ moiety feature in the ^1^H NMR spectrum (see ESI†). The solution-phase magnetic moment (Evans NMR, DFB, 243–298 K, Ar) was found to be 1.75 *μ*_B_, and the X-band ESR spectrum (PhMe/DFB glass, Ar, 40 K) revealed a rhombic signal (*g*_1_ = 2.483, *g*_2_ = 2.234, *g*_3_ = 1.985) with an isotropic *g*-value (*g*_iso_) of 2.23, [*g*_iso_ = (*g*_*x*_ + *g*_*y*_ + *g*_*z*_)/3; Fig. 1(c)]. These data are consistent with a low-spin (*S* = 1/2, d^7^) Fe(i) centre. The strong similarity between the X-band ESR spectrum of a powdered sample of [**1**]^+^[BArF4]^–^ (Ar, 1 bar, 40 K) and solution measurements implies that [**1**]^+^ is virtually isostructural in solution and the solid state.

Dissolution of [**1**]^+^[BArF4]^–^ in N_2_-saturated solvents (1 atm; DFB or THF) afforded forest-green solutions at room temperature, which became pale yellow upon cooling to –30 °C; either heating these solutions above room temperature, or degassing with Ar, resulted in the rapid reappearance of the characteristic blue colour of [**1**]^+^[BArF4]^–^. An IR active *ν*_NN_ stretch at 2067 cm^–1^ confirmed coordination of N_2_ to [**1**]^+^ to form [**1**·N_2_]^+^, which is intermediate in value between those seen for **1**·N_2_ and the related Fe(ii) [*trans*-Fe(H)(N_2_)(depe)_2_]^+^ (1975 cm^–1^ and 2102 cm^–1^ respectively);^16^ this trend may be readily accounted for by decreased Fe → N_2_ π-backbonding as the oxidation state increases.

**Fig. 2:**
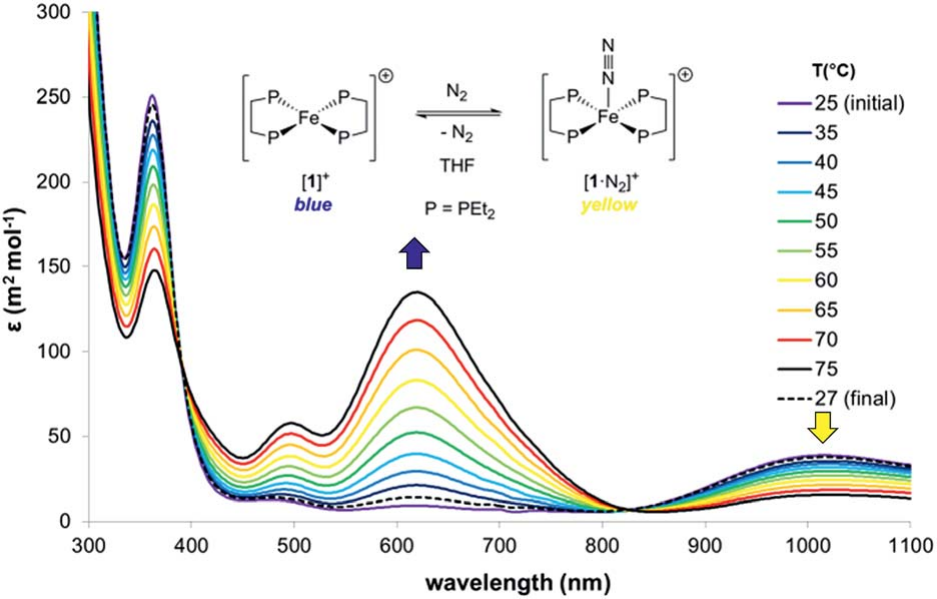
Variable-temperature UV-vis spectra of [**1**]^+^[BArF4]^–^ in THF under 1 atm. N_2_ ([BArF4]^–^ counterions omitted). Thermodynamics of N_2_ binding: Δ*H*^o^ = –13.1(1) kcal mol^–1^, Δ*S*^o^ = –27.6(1) cal K^–1^ mol^–1^; *K*_298_ = 4.1(3) × 10^–3^ M^–1^.

The thermodynamics of N_2_ coordination were obtained from variable temperature UV-vis spectroscopy by monitoring the concentrations of [**1**]^+^ and [**1**·N_2_]^+^*via* their absorption features (*λ*_max_ (nm) = 618 and 1018, respectively; Fig. 2).^17^ N_2_ association with [**1**]^+^ is accordingly found to be exoergic (Δ*G*_298_ = –4.9(1) kcal mol^–1^) with, as expected, a favourable enthalpy (Δ*H*^o^ = –13.1(1) kcal mol^–1^) and an unfavourable entropy (Δ*S*^o^ = –27.6(1) cal K^–1^ mol^–1^) contribution; these values compare well with those for N_2_ binding by (P_3_B)Co [P = *o*-(P*i*Pr_2_)_2_C_6_H_4_; Δ*H*^o^ = –13.9(7) kcal mol^–1^, and Δ*S*^o^ = –32(5) cal K^–1^ mol^–1^], which also produces an *S* = 1/2 dinitrogen complex.^18^,^19^

The X-band ESR spectra of rapidly freeze-quenched N_2_-saturated DFB or THF solutions (40 K) of [**1**]^+^[BArF4]^–^ revealed a pseudo-axial signal which differs markedly from the rhombic signal characteristic of the N_2_-free complex (*g*_iso_ = 2.07; simulated g tensors = 2.0014, 2.0922, 2.125). Additionally, hyperfine coupling to the four ^31^P nuclei was resolved for the THF glass giving *A*(^31^P) = 62.7(1) MHz (Fig. S5†), which is very similar to *A*_iso_(^31^P) previously obtained for [**1**·N_2_]^+^[TfO]^–^ [66.2(2) MHz].^8^

**Fig. 3:**
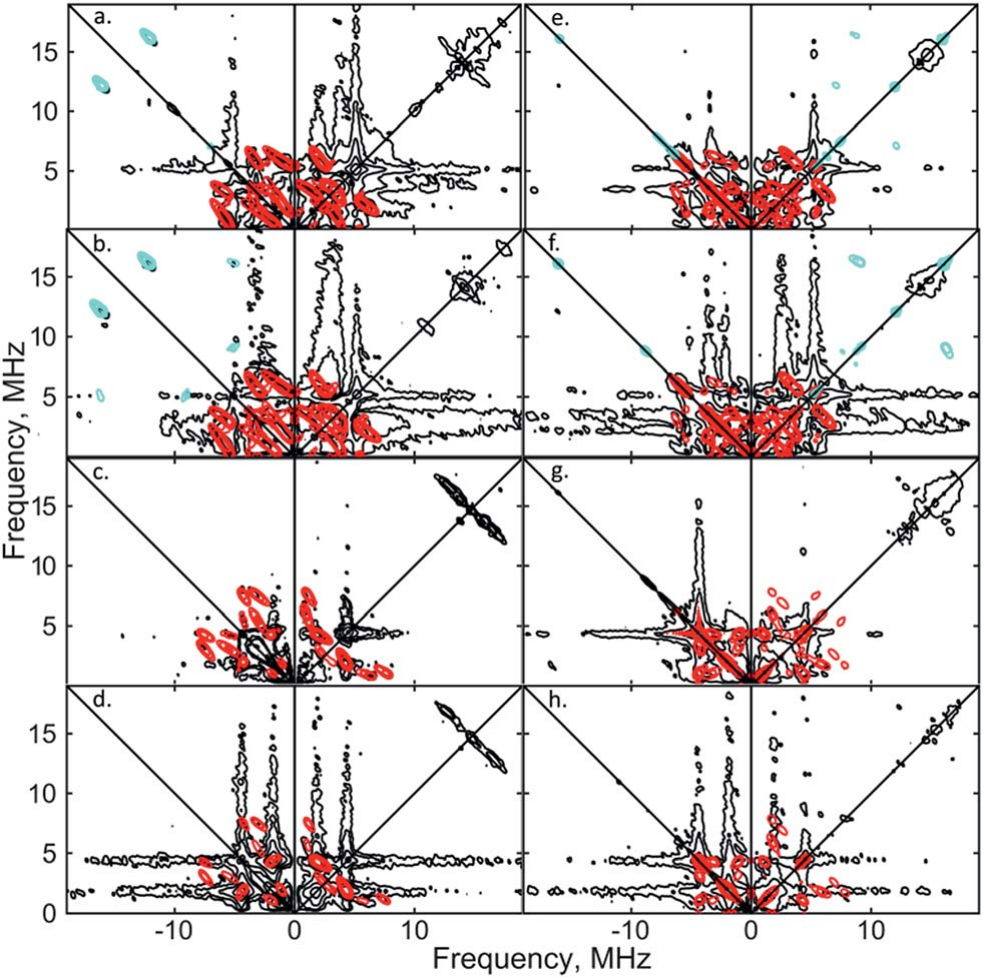
X-band 2D ESEEM spectra of 2.5 mM [**1**·^14^N_2_]^+^[BArF4]^–^ in THF, with standard 4-pulse HYSCORE (panels (a–d)), and DONUT HYSCORE^21^ (panels (e–h)), at two fields (3471 G: (a, b, e and f); 3800 G: (c, d, g and h)) and two *τ* values (200 ns: (a, c, e and g); 132 ns: (b, d, f and h)) while *τ*_2_ = 800 ns for DONUT. The samples were frozen glasses at 20 K and measured with pulse lengths of π/2 = 8 ns and π = 12 ns, initial variable delays of 100 ns, and a time step of 20 ns over 200 points in both axes. The microwave frequency was 9.7449 GHz. Experimental data is in black, while separate simulations of two ^14^N are overlaid in red and cyan.

Similar spectroscopic observations have been described for the Fe(i) complex [Fe(DMeOPrPE)_2_(N_2_)]^+^ (DMeOPrPE = R_2_PCH_2_CH2PR_2_; R = CH_2_CH_2_CH_2_OMe), which were attributed to an equilibrium between yellow [Fe(DMeOPrPE)_2_(N_2_)]^+^ and a purple N_2_-bridged bimetallic *S* = 1 species {[Fe(DMeOPrPE)_2_]_2_(μ-N_2_)}^2+^; the latter is favoured at higher temperatures and/or low *p*(N_2_).^20^ We noted that comparable optical absorptions were observed for [**1**]^+^ and [**1**·N_2_]^+^ (see Table S1, ESI†), and accordingly 2D-ESR (HYSCORE; HYperfine Sub-level CORrElation) experiments were performed on equivalent ^14^N_2_- and ^15^N_2_-saturated PhMe/DFB (7 : 1) solutions of [**1**]^+^[BArF4]^–^, to fully ascertain the solution-phase coordination mode of N_2_ to [**1**]^+^. As shown in Fig. 3, two ^14^N signals were clearly detected at the perpendicular field position, in panels a, b, e, f.

The proximal nitrogen, ^14^N_p_, gives intense double quantum dq, dq correlation peaks in the (–, +) quadrant corresponding to the ^14^N directly bound to Fe(i) centre, leading to peaks at (∓16.2, ±12.2) MHz and, from eqn (6), *A*_iso_(^14^N_p_) may correspondingly be calculated as 14.3 (±0.1) MHz.^22^ For the distal nitrogen, ^14^N_d_, field-dependent ^15^N_2_ simulations of 4-pulse HYSCORE provide *A*_iso_(^14^N_d_) = 4.4 MHz (seen in ESI, Fig. S7†). A clearly-resolved quadrupole interaction of ^14^N_p_ reveals a very small asymmetry *η* ∼ 0, which compares well with sp-hybridized ^14^N found in N≡N and [C≡N]^–^.10*a* For the ^14^N_d_ (red in Fig. 3), it was found that *K* = 0.9 MHz. In the case of the ^14^N_p_, the data was insufficient for a more precise determination, and the quadrupole values of ^14^N_d_ were used as an approximation. For ligand atoms directly bonded with covalent character, a point-dipole model can be inaccurate; for ^14^N_d_*T* = 1.2 MHz, and for ^14^N_p_ the simulation value was *T* = 0.6 MHz. To confirm that both ^14^N signals derive from the same molecule (considering that no large ^15^N hyperfine coupling was observed) close examination of the dq, dq peaks at 3471 G reveals weak multinuclear combination frequency peaks such as {N_p_^*α*^(dq), N_p_^*β*^(dq), N_d_^*β*^(dq)} (seen in Fig. S7†), a signal class previously reported by Stich *et al.* for Mn_2_(iii/iv) Catalase.^23^ It is notable that no ‘half-field’ resonance (*g* ≈ 4) was observed in the CW-ESR experiments, which would be expected for the hypothetical triplet (*S* = 1) {[Fe(depe)_2_]_2_(μ-N_2_)}^2+^ due to a formally forbidden Δ*M*_s_ = 2 transition, as has been documented for other transition metal diradicals.^24^ Collectively alongside other spectroscopic data, these observations strongly support the solution-phase assignment of [**1**·N_2_]^+^[BArF4]^–^ as [Fe(depe)_2_(η^1^-N≡N)]^+^[BArF4]^–^.

**Fig. 4:**
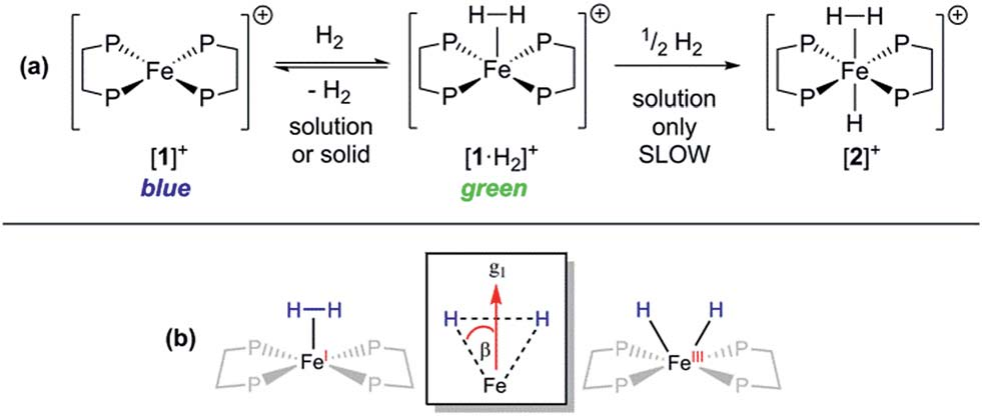
(a) H_2_ coordination by [**1**]^+^[BArF4]^–^. (b) Schematic showing angle *β* between *g*_–1_ principal axis and Fe–H bond vectors, relevant to electron-^1^H dipolar coupling for alternative [**1**·(H_2_)]^+^ and [**1**(H)_2_]^+^ structures, from reaction of [**1**]^+^ and H_2_. P = PEt_2_; [BArF4]^–^ counteranions omitted.

Given that {TM(σ-N_2_)} and {TM(σ-H_2_)} fragments are related by the same VE count, numerous diamagnetic metal–ligand platforms have been shown to interconvert these species under N_2_ and H_2_ mixtures;^25^ however, analogous open-shell examples are extremely rare.^26^ Furthermore, only two thoroughly characterised paramagnetic dihydrogen ligand complexes have been reported to date: (P_3_B)Co(H_2_)^18^ and (P_3_Si)Fe(H_2_);^7^ both of these are idealised *C*_3_ symmetric, trigonal bipyramidal *S* = 1/2 complexes. In spite of the different coordination geometry and cationic charge, admission of H_2_ (1 atm.) to DFB or THF solutions of [**1**]^+^[BArF4]^–^ demonstrated clear reaction (Fig. 4(a)), as evidenced by an immediate colour change to pale green (*λ*_max_ = 850 nm, *ε*_max_ ≈ 19 m^2^ mol^–1^) and the appearance of a new near-axial ESR signal (X-band, PhMe/DFB, 40 K; *g* = [2.000, 2.085, 2.160], *g*_iso_ = 2.08, Fig. S6†), which displays more pronounced hyperfine splitting for *g*_2_ and *g*_3_ than seen for [**1**·N_2_]^+^[BArF4]^–^.^27^ The most plausible identities of this species are the Fe(i) adduct [Fe(depe)_2_(σ-H_2_)]^+^ ([**1**·H_2_]^+^) or the Fe(iii) oxidative addition product [Fe(depe)_2_(H)_2_]^+^ ([**1**(H)_2_]^+^).^28^ Using the DFT-optimised structures of [**1**·H_2_]^+^ and [**1**(H)_2_]^+^ (*vide infra*), the angle (*β*) between the *g*_1_ principal axis and the Fe–H vectors was calculated to be 16° and 31°, respectively.

**Fig. 5:**
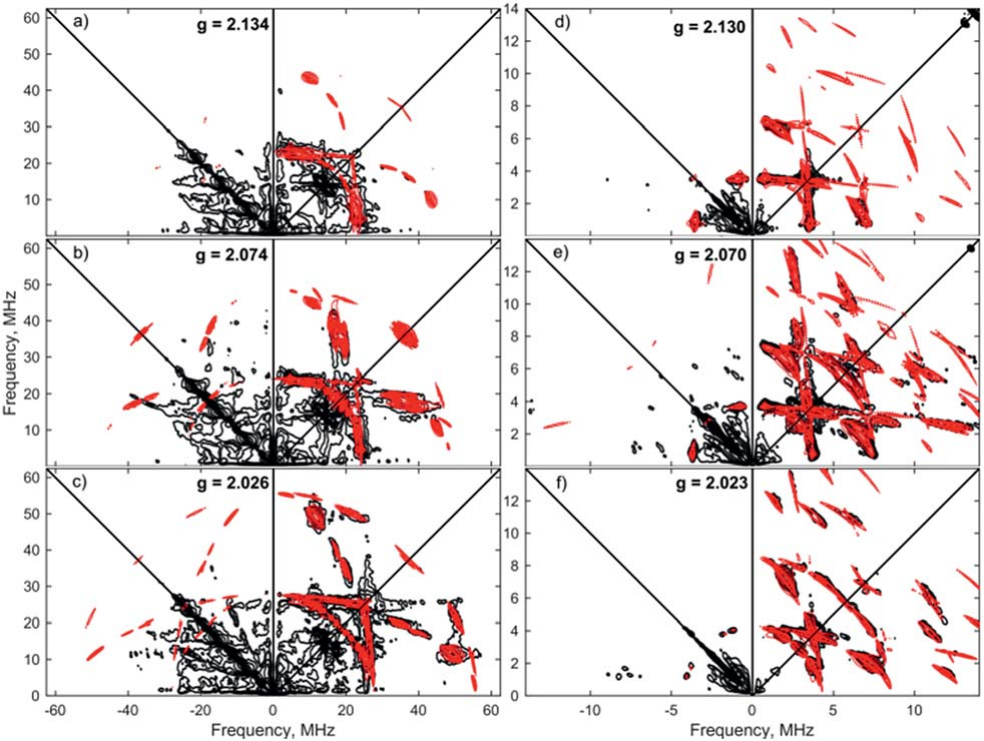
X-band 4-pulse HYSCORE spectra of [**1**·^1^H_2_]^+^[BArF4]^–^ (panels (a–c)) and [**1**·^2^H_2_]^+^[BArF4]^–^ (panels (d–f)) at 2.5 mM in 7 : 1 PhMe : DFB, each collected at three field positions as indicated by *g*-value. The samples were frozen glasses at 20 K and measured with pulse lengths for ^2^H of π/2 = 12 ns and π = 8 ns, *τ* = 204 ns, initial variable delays of 100 ns and a time step of 20 ns over 300 points in both axes. The pulse lengths for ^1^H were π/2 = 8 ns and π = 12 ns, *τ* = 144, 140, 132 ns (top to bottom), initial variable delays of 100 ns and a time step of 8 ns, and 200 points in both axes. The microwave frequency was 9.7411 and 9.7583 GHz, respectively. Experimental data is in black, while simulations are overlaid in red.

Orientation-selective ENDOR has previously been used to differentiate between Fe(H_2_) and Fe(H)_2_ formulations using electron–^1^H dipolar hyperfine coupling interactions, where only small *β* angles (<15°; see Fig. 4(b)) were able to reproduce the observed experimental lineshape.^7^,^29^ The 1 : 2 : 1 hyperfine seen at the five negative ^31^P peaks of *g*_min_ in CW-ESR of Fig. S6,† was interrogated more closely with H_2_/D_2_-saturated solutions of [**1**]^+^[BArF4]^–^ using ^1^H Davies ENDOR (Electron-Nuclear DOuble Resonance) and both ^1^H & ^2^H HYSCORE experiments (see Fig. 5). Fits to data from both techniques used two H hyperfine interaction values, for ^1^H *A*(^1^H) = [–17.99, –19.93, 26.58]/MHz, while the values were scaled by the nuclear *g*-factor ratio *g*_n_(^2^H)/*g*_n_(^1^H) = 0.1535 for the ^2^H HYSCORE simulation, revealing a dipolar component of *T* = 15.2 MHz. The Fe–H distance, *r*_FeH_ (Å), can thus be obtained from *r*_FeH_ (Å) = (Å) = ∛((*ρ*_(Fe)_79.06/*T*), using a spin density of *ρ*_(Fe)_ ∼ 0.83 as remainder of the large isotropic ^31^P hyperfine interactions;^30^,^31^ this coupling is consistent with an Fe–H bond distance of 1.64 Å. Considering the spin density at Fe and the coordinating ligand atoms and DFT coordinates (*vide infra*), eqn (3) was used to fit an angle of 2*β* = H–Fe–H, *β* = 5.5°, using the empirical principal dipolar values [–14.2, –16.2, 30.4] MHz (written as [–*T*(1 – *δ*), –*T*(1 + *δ*), 2*T*], with *δ* as the rhombicity parameter); this value is in excellent agreement with that found for (P_3_Si)Fe(H_2_) (*β* = 6°),^7^ which was interpreted as indicative of partial rotational averaging of the dipolar interaction. However, the angle *β* = 5.5° includes spin density on opposing ^1,2^H, and Morris *et al.*, has shown that rotational motion can reduce H–H dipolar interactions by up to a factor of four, implying that *β* = 5.5° should be considered an upper limit.^32^

**Fig. 6:**
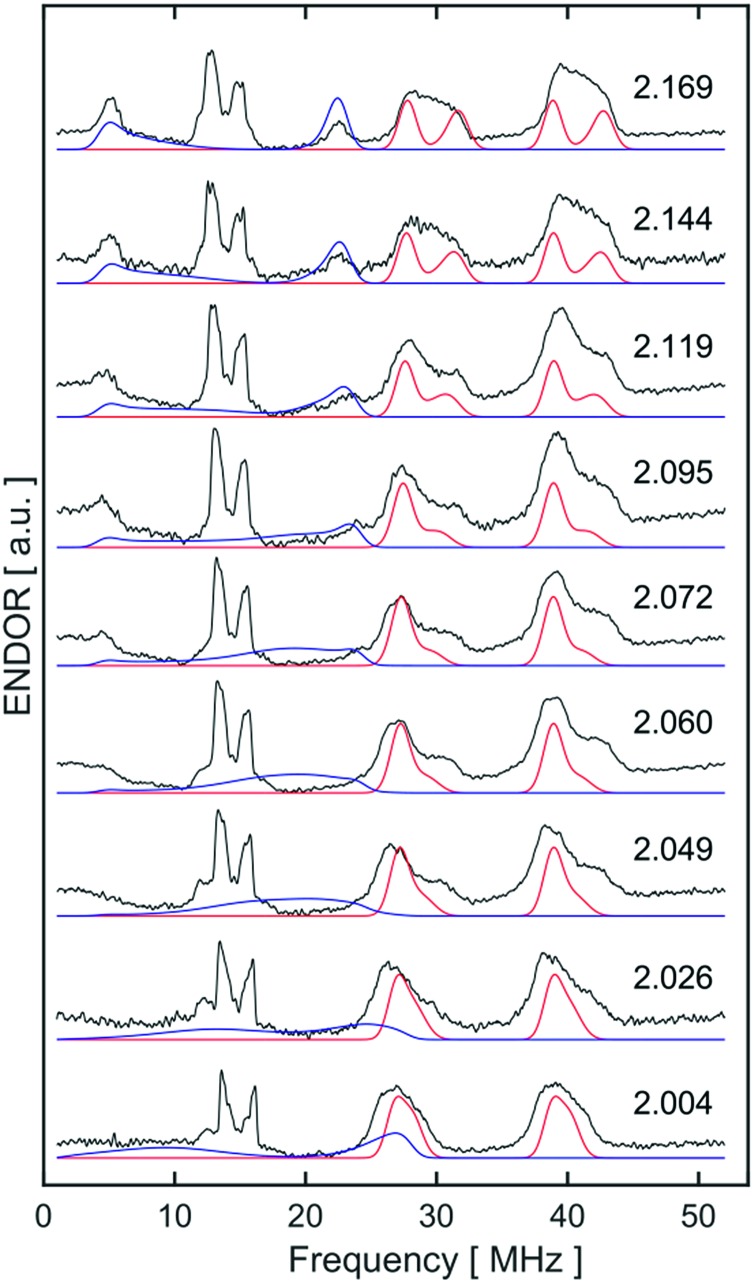
X-band Davies ENDOR spectra of [**1**·^1^H_2_]^+^[BArF4]^–^ at 2.5 mM in 7 : 1 PhMe : DFB, collected at nine field positions as indicated by *g*-value along the right side. The sample was a frozen glass at 20 K and measured with pulse lengths of π/2 = 40 ns and π_inv_ = π = 80 ns, π = 300 ns, RF pulse = 16 μs, and 1 μs before and 2 μs after the RF pulse, with stochastic frequency stepping. The microwave frequency was 9.7621 GHz. Experimental data is in black, while simulations of ^31^P values are red, and simulations of ^1^H are in blue (as discussed in main text).

Simulations of ENDOR data for [**1**·H_2_]^+^ in Fig. 6 are consistent with the DFT structure (*vide infra*), in having two classes of ^31^P hyperfine interaction, *a*_iso_(^31^P) = 69.3 MHz of P in-plane orthogonal to the Fe(i)–H_2_ bond and *a*_iso_(^31^P) = 66.3 MHz for the P bent out-of-plane; furthermore the P–Fe–P depe ligand angle of 85.5° suggests that the symmetry lies closer to 2-fold than 4-fold. Correspondingly, these results provide strong evidence that [**1**·H_2_]^+^ is best described as a non-classical σ-H_2_ adduct.

**Fig. 7:**
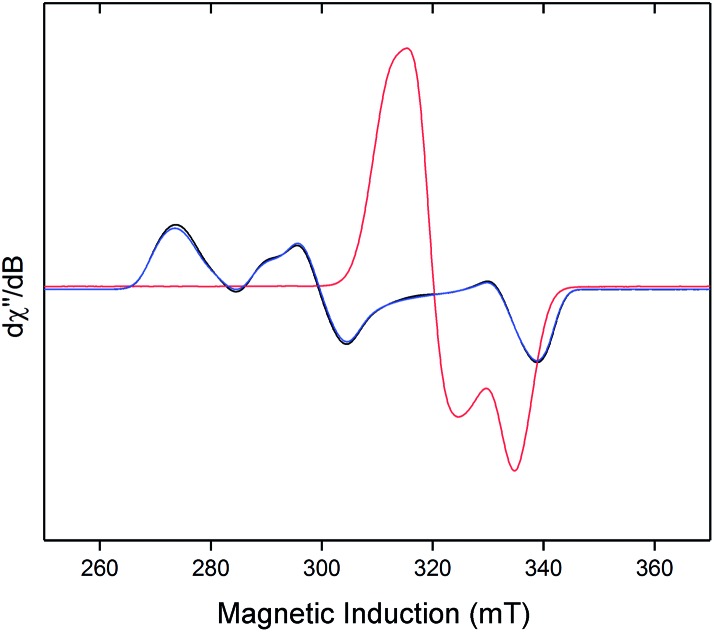
Reversible binding of H_2_ by [**1**]^+^[BArF4]^–^ in the solid-state: CW X-band ESR spectra of powdered [**1**]^+^[BArF4]^–^ under Ar (black), followed by evacuation and subsequent admission of H_2_ (1 bar, [**1**·H_2_]^+^[BArF4]^–^, pink), and sequential evacuation to 10^–3^ mbar and backfill with Ar (blue). All spectra recorded at 40 K.

Freshly-prepared solutions of [**1**·H_2_]^+^ are sufficiently stable for detailed *in situ* characterisation and, as with solutions under N_2_, vacuum/Ar-degassing resulted in regeneration of spectroscopic signals attributed to [**1**]^+^, demonstrating reversible coordination of H_2_. Nevertheless, attempts to obtain thermochemical information for H_2_ binding were unfortunately frustrated by slow and irreversible formation of the Fe(ii) trishydride *trans*-[Fe(depe)_2_(H)(H_2_)]^+^, [**2**]^+^.^33^ Appreciating that ligation of N_2_ or H_2_ to [**1**]^+^ results in minimal deformation of the [Fe(depe)_2_]^+^ core,^34^ we speculated that binding of these gases might also be reversible in the solid state, as in solution. Gratifyingly, admission of either N_2_ or H_2_ to powdered samples of [**1**]^+^[BArF4]^–^ led to comparable colour changes (deep blue to yellow or green, respectively), consistent with clean conversion to [**1**·(N_2_/H_2_)]^+^[BArF4]_(s)_^–^. ESR spectra closely matched those obtained in solution, strongly suggesting that the geometry of the [Fe(depe)_2_]^+^ core is preserved in both phases upon N_2_ and or H_2_ coordination. Crucially, removal of N_2_/H_2_ from these samples under vacuum led to complete restoration of the original ESR signal of [**1**]^+^ (Fig. 7 and S2†), confirming that binding is fully reversible (over multiple cycles) in the solid state; importantly, no loss in signal intensity was observed (which would be expected from formation of diamagnetic [**2**]^+^) under H_2_. Furthermore, ready exchange of the N_2_ ligand for H_2_ is achieved *via* simple evacuation of N_2_ and replacing with H_2_, and *vice versa*.

ESR spectra of [**1**]^+^[BArF4]^–^ obtained in the presence of a single equivalent of N_2_ or H_2_ allow for a quantitative comparison of the binding affinities of the two gases; the ratio [**1**·L]^+^/[**1**]^+^ (L = N_2_ or H_2_; determined by signal intensity at 303 K) is significantly larger for H_2_ (44) than N_2_ (0.15), revealing that binding of the former is almost 300 times more favourable at ambient temperature.^35^ For comparison, (P_3_B)Co(L) (L = N_2_, H_2_) are in rapid dissociative equilibrium in solution under similar conditions, whereas (P_3_Si)Fe(L) species require several days to interconvert (proposed to proceed *via* an associative mechanism involving partial dechelation of the P_3_Si ligand);^7^,^18^ both demonstrate a preference for H_2_ binding, albeit to differing degrees (*K*_H_2__/*K*_N_2__ ≈ 2 and 50 for Co and Fe, respectively). This difference in exchange kinetics was postulated to result from the poorer π-backbonding capability of Co *vs.* Fe which leads to weaker M–L interactions. Hence it is plausible that the ready reversibility of N_2_ and H_2_ exchange for [**1**]^+^ relative to (P_3_Si)Fe could also be due (in part) to poorer π-donation from the former, by virtue of its cationic charge, which is also manifest in the higher *ν*_NN_ stretch value of the former (2067 *vs.* 2003 cm^–1^).

## Computational calculations

In order to probe the structure and electronic properties of [**1**]^+^, [**1**·N_2_]^+^, and [**1·**H_2_]^+^, density functional calculations were carried out using the ADF program suite version 2014.1.^36^ The Slater-type orbital (STO) basis sets were of triple-*ζ* quality augmented with a one polarization function (ADF basis TZP). Core electrons were frozen (C, N 1s; Fe 2p) in our model of the electronic configuration for each atom. The local density approximation (LDA) by Vosko, Wilk and Nusair (VWN)^37^ was used together with the exchange correlation corrections of Becke and Perdew (BP86).^38^

**Fig. 8:**
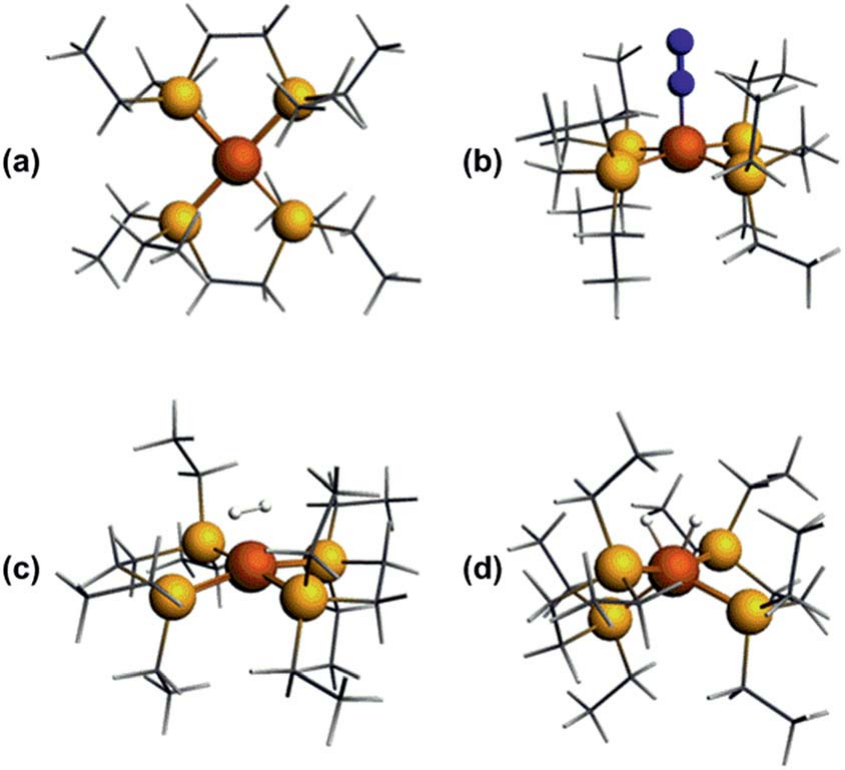
DFT-optimised structures of (a) [**1**]^+^, (b) [**1**·N_2_]^+^, (c) [**1**·H_2_]^+^, (d) [**1**(H)_2_]^+^.

Optimized geometries were ascertained as local minima *via* frequency calculations. Geometry optimisation of base-free [**1**]^+^ with *S* = 1/2 resulted in a *D*_2_ structure consistent with X-ray crystallographic data, with an angle of approximately 7° between the two iron-ligand Fe(PP) coordination planes (Fig. 8(a)); fixing the spin state to *S* = 3/2 showed the alternative high-spin structure to be some 0.77 eV (approximately 17.8 kcal mol^–1^) higher in energy. Geometry optimisation of [**1**·N_2_]^+^ results in a structure of *C*_2_ symmetry with a slightly increased angle between the two Fe(PP)_2_ planes of 19° (Fig. 8(b)). The coordination geometry around Fe is square-based pyramidal with P–Fe–N_α_ angles of 94.8° and 95.6°. The calculated stretching frequency for the bound N_2_ ligand was 2059 cm^–1^, which is in good agreement with experiment (2067 cm^–1^). Geometry optimisation of [**1**·H_2_]^+^ (Fig. 8(c)) gave a σ-complex with a H–H bond length of 0.899 Å (*cf.* free H_2_: 0.74 Å)^6^ and an average Fe–H distance of 1.61 Å, which correlates well with 2D-ESR data. An alternative [**1**·(H)_2_]^+^ isomer (Fig. 8(d)), corresponding to the product of H_2_ oxidative addition and containing two well-separated hydride ligands (H···H = 1.57 Å and Fe–H distances of 1.51 Å), could also be located, albeit 12 kcal mol^–1^ higher in energy than [**1**·H_2_]^+^ (see Table S2 in ESI for further details†).

**Fig. 9:**
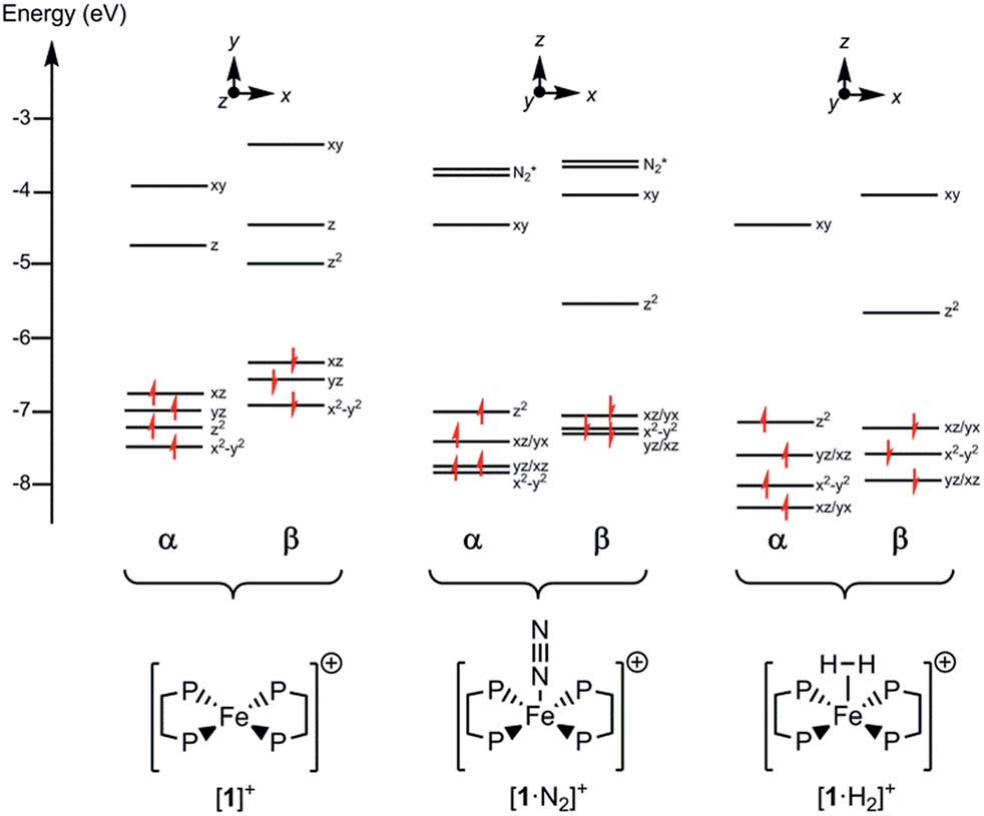
Electronic energy levels and their occupancy for [**1**]^+^, [**1**·N_2_]^+^ and [**1**·H_2_]^+^. The principal Fe character is indicated.

Since all compounds had unpaired spins the DFT calculations were unrestricted, with different orbitals for *α* and *β* spins. The energies of the *α* spin electrons, of which there are more, tended to be lower than those of the *β* spin electrons in corresponding orbitals because of exchange stabilization (Fig. 9).

**Fig. 10:**
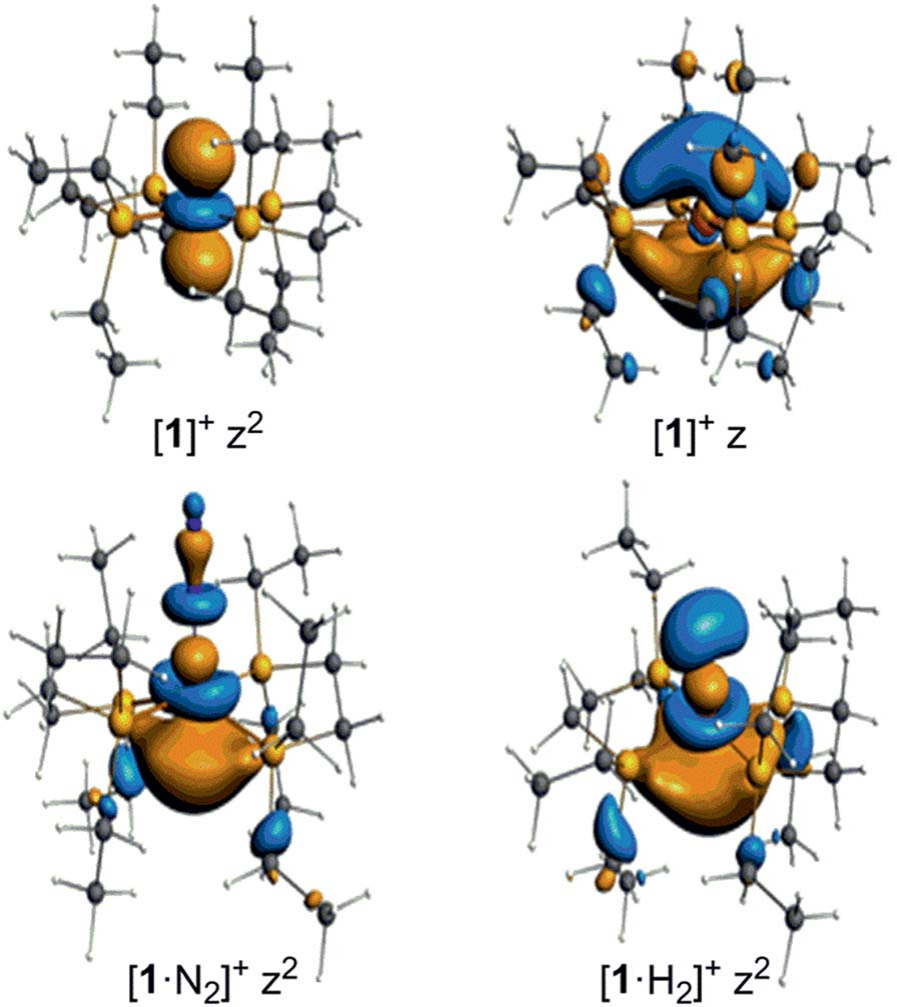
Selected Kohn–Sham isosurfaces for [**1**]^+^, [**1**·N_2_]^+^ and [**1**·H_2_]^+^ showing the *z*^2^ occupancy of the unpaired electrons and the virtual orbital for [**1**]^+^ of primarily 4p_*z*_ character.

The unpaired electron in the three cases, [**1**]^+^, [**1**·N_2_]^+^, and [**1·**H_2_]^+^, occupies an orbital of primarily d(*z*^2^) character; in the case of [**1**·N_2_]^+^ and [**1**·H_2_]^+^ this is also hybridized with the Fe 4p(*z*) orbital (Fig. 10), and is antibonding with respect to the coordinated N_2_ or H_2_, thus explaining the weak association of these ligands to the [Fe(depe)_2_]^+^ core. Of particular interest is the virtual orbital (*z*) of [Fe(depe)_2_]^+^, the isosurface of which is shown in Fig. 10; its AO composition is predominantly Fe 4p(*z*) and P 3p.

Time-dependent DFT (TDDFT) was used to calculate the electronic absorption spectra of [**1**]^+^, [**1**·N_2_]^+^ and [**1**·H_2_]^+^ (see Table S3 and Fig. S15 in the ESI†). The numerical agreement with experiment is only moderate, with the bands being calculated at higher energy than measured experimentally. All three compounds have as their lowest energy bands d–d transitions, with a *β* spin electron being excited into the hole in the d(*z*^2^) orbital. However those bands of [**1**]^+^ have effectively zero oscillator strength on account of its high symmetry. The d–d transitions are of longer wavelength for [**1**]^+^ than for [**1**·N_2_]^+^ and [**1**·H_2_]^+^, which fits with the smaller HOMO–LUMO gap in the former (see Fig. 9). The subsequent set of bands calculated at 587, 548 and 503 nm for [**1**]^+^ fit well with the features found at 500 and 618 nm experimentally; they are of significantly higher oscillator strength than the d–d bands of [**1**·N_2_]^+^ and [**1**·H_2_]^+^ and correspond to excitation from the occupied 3d orbitals into the Fe 4p_*z*_ orbital. In [**1**·N_2_]^+^ and [**1**·H_2_]^+^, the corresponding band is absent as a consequence of ligand binding along the *z* axis, hence their respective absorption spectra have no analogous feature. Thus, in spite of the lack of numerical agreement for the d–d bands, many features of the calculated spectra give a good account of those observed.

## Conclusions

In conclusion, we have synthesised and fully characterised an open-shell, cationic Fe(i) complex, [**1**]^+^[BArF4]^–^, and demonstrated the readily reversible coordination of N_2_ and H_2_ to the *S* = 1/2 Fe centre. Remarkably, this facile exchange between N_2_ and H_2_ coordination occurs in either solution or solid states under ambient conditions, likely due to the very small structural change in transitioning between square planar [**1**]^+^ and square-based pyramidal [**1**·(N_2_/H_2_)]^+^ species. Furthermore, [**1**·H_2_]^+^[BArF4]^–^ is a rare example of a well-defined paramagnetic σ-H_2_ complex, as corroborated by ESR spectroscopy and DFT calculations. These results reveal that reversible coordination of these small molecules to open-shell complexes is neither restricted to neutral compounds nor *C*_3_ molecular symmetries, which until now, have been the only known examples. Given the importance of Fe in catalytic N_2_ fixation, this work is of significant relevance to the ongoing development and investigation of well-defined transition metal catalysts for N_2_ reduction, mediated by H^+^/e^–^ sources or (ideally) H_2_ as the terminal reductant.

## Conflicts of interest

There are no conflicts to declare.

## Supplementary Material

Supplementary information

Crystal structure data
