# Supplementary material for: Reversible coordination of N2 and H2 to a homoleptic S = 1/2 Fe(i) diphosphine complex in solution and the solid state
Source: Chem Sci. 2018 Jul 18;9(37):7362–9. doi: 10.1039/c8sc01841c (PMC6237127; doi:10.1039/c8sc01841c)
Supplement: Supplementary file 1 [file SC-009-C8SC01841C-s001.pdf]

Electronic Supplementary Material (ESI) for Chemical Science.
This journal is © The Royal Society of Chemistry 2018

Electronic Supplementary Information (ESI)

Reversible coordination of N_2 and H_2 to a homoleptic S = ½ Fe(I) diphosphine complex in solution and the solid state

Laurence R. Doyle,[a] Daniel J. Scott,[a] Peter J. Hill,[a] Duncan A. X. Fraser,[a] William K. Myers,*[b] Andrew J. P. White,[a] Jennifer C. Green,[b] and Andrew E. Ashley*[a]

[a] *Department of Chemistry, Imperial College London, Exhibition Road, South Kensington, London SW7 2AZ, UK. E-mail: a.ashley@imperial.ac.uk Tel: +44 0(20) 759 45810.*

[b] *Inorganic Chemistry Laboratory, University of Oxford, Oxford OX1 3QR, UK.*

Contents:

1. Experimental details 1
2. NMR spectroscopy 6
3. ESR spectroscopy 7
4. IR spectroscopy 12
5. UV-vis spectroscopy 13
6. Computational data 17
7. X-ray diffraction data for **[1]$^+$[BAr$^F_4$]$^-$** 26
8. References 28

1. Experimental details

General considerations

All chemical manipulations were performed under a N_2 or Ar atmosphere either using standard Schlenk-line techniques or a MBraun Labmaster DP glovebox, unless stated otherwise. H_2 (BOC, 5.5 Research Grade) was dried by passage through a NANOCHEM® WA-500 OMX™ purifying column. Solvents were purchased from VWR: pentane and hexane were dried using an Innovative Technology Pure Solv™ SPS-400; THF, Et_2O and 1,4-dioxane (Sigma-Aldrich) were distilled from dark green Na/fluorenone indicator; 1,2-difluorobenzene (DFB; Fluorochem) was distilled from CaH_2; *tert*-butyl methyl ether (TBME; Sigma-Aldrich) was dried over 4 Å molecular sieves. Solvents were degassed by thorough sparging with N_2 or Ar gas and stored in gas-tight ampoules: pentane, hexane and Et_2O were stored over a K mirror; DFB and TBME were stored over 4 Å molecular sieves. Deuterated solvents were freeze-pump-thaw degassed, and stored in gas-tight ampoules over 4 Å molecular sieves: C_6D_6 (Sigma-Aldrich, 99.5 atom % D), THF-d_8 (Sigma-Aldrich, 99.5 atom % D). Mg powder, 1,2-dibromoethane, and Cp_2Fe were purchased from Sigma-Aldrich; the latter was purified by sublimation and recrystallisation from cold pentane. Fe(depe)$_2$N$_2$, (**1**·N$_2$),[1] *trans*-Fe(depe)$_2$(Cl)$_2$,[2] *trans*-Fe(depe)$_2$(H)(Cl),[3] [Cp$_2$Fe][BAr$^F_4$] (Ar^F = 3,5-(CF$_3$)$_2$C$_6$H$_3$),[4] and Na[BAr$^F_4$][5] were prepared according to literature procedures.

NMR spectra were recoded using Bruker AV-400 (400.4 MHz) spectrometers. Chemical shifts, δ, are reported in parts per million (ppm). ^{1}H chemical shifts are given relative to Me_4Si and referenced internally to the residual proton shift of the deuterated solvent employed. ^{31}P chemical shifts were referenced (δ = 0) externally to 85% H_3PO_4 (aq). ^{1}H and ^{31}P NMR spectra of solutions prepared in non-deuterated solvents incorporate an internal reference capillary containing a solution of ca. 0.1 M PPh_3 in C_6D_6 and are referenced to residual C_6D_5H and PPh_3 (δ = –5.3) resonances, respectively. Air or moisture sensitive samples were prepared inside a glove-box using NMR tubes fitted with J. Young valves. The paramagnetic susceptibility of solution-phase samples was determined using the Evans NMR method.[6,7]

Infrared (IR) spectra were recorded using a Perkin Elmer FT-IR Spectrum GX spectrometer. Samples were measured as either KBr pellets or solutions. To form a KBr pellet, the sample was ground with KBr (Sigma, FT-IR grade), and subsequently pressed in an air-tight Specac® die using a Specac® manual hydraulic press. Solutions were recorded using an air tight Specac® Omni Cell™, which was loaded with a ca. 0.1 M solution *via* syringe inside the glove-box.

Electronic spectra were recorded using a Perkin Elmer Lambda 20 UV-visible spectrophotometer. Samples were prepared inside the glove-box using a quartz cuvette with an optical path length of 1 cm and fitted with a J. Young valve. Variable-temperature UV-vis spectra were recorded using a Unisoku CoolSpeK UV USP-203-B accessory. The solubility of N_2 in THF (x_{N2} = 5.21 x 10^{-4}; 6.4 mM at 298 K and 1 atm.) for the variable-temperature equilibrium measurements was obtained from the literature.[8]

ESR data were collected at the Centre for Advanced Electron Spin Resonance (CAESR), of the Department of Chemistry, University of Oxford. The CW-EPR spectrometer for cryogenic conditions (40-85 K) for frozen solutions in J. Young tubes and flame-sealed pulsed EPR tubes was a Bruker BioSpin EMXmicro with an EMX Premium bridge, an Oxford Instruments ESR-900 cryostat, an Oxford Instruments ITC-503s temperature controller, and a Bruker BioSpin Super High-Q resonator (ER 4123-SHQE-W), which operates in the cylindrical TE_{011} mode. The CW-EPR spectrometer for elevated temperatures of solid films in the bottom J. Young tubes (3-4 cm height on wall) was a Bruker BioSpin ER4001100 EMX with a Bruker W1702025 MR G 0141 Gunn bridge, a Bruker liquid N2 heater cryostat, a Bruker VT-1000 temperature controller calibrated by an external K-type thermocouple of the sample tube dimensions for reproducing flow conditions, and a Bruker BioSpin ER4119HS/W, which operates in the cylindrical TE_{011} mode. To prepare samples for pulsed EPR, bottom portion of J. Young tubes of 3.8 mm O.D., containing ca. 150 μL of 2.5 mM frozen analyte solution, were flame sealed under vacuum of ~ 3.5x10^{-2} mBar, excising the bottom section of about 19 cm length, which was stored at 77 K and not melted prior to X-band pulsed EPR measurements. HYSCORE and ENDOR were acquired with $TE_{01\delta}$-mode cylindrical sapphire EN4118X-MD4EN-W1 dielectric resonators, with Bruker BioSpin E680 EleXSys and E580 EleXSys II spectrometers with Oxford Instruments CF935 cryostats at a temperature of 10 K. EPR Simulations employed the EasySpin 5.1.0 simulation toolkit functions for MatLab (version 8.6.0, R2015b, Mathworks, Inc.)[9]

Uncertainty in hyperfine simulations estimated as +/- 0.5 MHz. For powder ESR experiments, a stock solution of [**1**]$^+$[BAr$^F_4$]$^-$ in DFB (25 mM) was prepared in an Ar-filled glovebox. For each experiment, 0.2 mL of stock solution (corresponding to 0.005 mmol [**1**]$^+$[BAr$^F_4$]$^-$) was transferred to a J. Youngs-adapted quartz ESR tube. The solvent was carefully removed *in vacuo* to leave a thin powder coating of the sample on the walls of the tube, and the tube subsequently back-filled to a pressure of 1 atm. with Ar, N_2 or H_2. Samples intended to contain precisely 1 eq. of N_2 or H_2 were initially prepared under Ar in same manner. The Ar was subsequently removed under vacuum, and 0.005 mmol of the appropriate gas was added through use of a Toepler pump.

Single crystal X-ray diffraction data for [**1**]$^+$[BAr$^F_4$]$^-$ were collected by Dr Andrew J. P. White with an Oxford Diffraction Xcalibur unit; the crystal was mounted on a glass fibre using perfluoropolyether oil and measured in a stream of N_2 at 173 K. The structure was solved by direct methods using SHELX.[10,11]

Elemental analyses were performed by Mr S. Boyer of the London Metropolitan University.

Synthesis and characterization.

[Fe(depe)$_2$]$^+$[BAr$^F_4$]$^-$, [1]$^+$[BAr$^F_4$]$^-$: Under an Ar atmosphere, [Cp_2Fe][BAr$^F_4$] (803 mg, 0.765 mmol) was dissolved in 5 mL of Et_2O and slowly added to a stirring solution of **1**·N_2 (400 mg, 0.806 mmol) in 5 mL of Et_2O. The resulting deep blue solution was stirred for 2 h at RT before volatiles were removed *in vacuo*. The remaining solid was rinsed with hexane (4 x 5 mL) and subsequently extracted into DFB, concentrated, and then layered with hexane. Slow diffusion of hexane into the DFB solution resulted in blue crystals which were collected by filtration, then rinsed with cold TBME (2 x 2 mL) followed by pentane (2 x 2 mL), before being dried *in vacuo* (935 mg, 87%).

Anal. Calcd. for $C_{52}H_{60}BF_{24}FeP_4$: C, 46.90; H, 4.54. Found: C, 46.82; H, 4.64.

^{1}H NMR (400.4 MHz, THF) *δ*: –0.54 and –1.77 (br s, overlapping), –21.30 (vbr s).

^{1}H NMR (400.4 MHz, DFB) *δ*: –0.33 and –1.67 (br s, overlapping), –21.12 (vbr s).

UV-vis: See Table S1.

[Fe(depe)$_2$(η^1-N$_2$)]$^+$[BAr$^F_4$]$^-$, [1·N$_2$]$^+$[BAr$^F_4$]$^-$: In a N_2-filled glove-box at 1 atm. pressure, [**1**]$^+$[BAr$^F_4$]$^-$ (13.3 mg) was dissolved in THF solvent (20 ml), from which 1 ml (overall concentration 0.5 mM) was used to fill a J. Youngs-adapted quartz cuvette, which was subsequently sealed and subjected to UV-vis measurements. For NMR experiments solutions of [**1**]$^+$[BAr$^F_4$]$^-$ in DFB (0.4 ml, 1 mM concentration) were prepared under Ar in a J. Youngs NMR tube, which were subsequently freeze-thaw-degassed three times with N_2 on a Schlenk-line manifold. The last backfill of N_2 (1 atm.) was subsequently sealed at liquid N_2 temperature, before being warmed to RT (internal pressure ~ 4

atm.). Solution ESR experiments were treated analogously to NMR experiments, except that the last backfill was at RT and hence the internal pressure was at 1 atm..

^{1}H NMR (400.4 MHz, DFB, 4 atm. N_2, 293 K) δ: –0.89 (br s).

IR (Et_2O, under N_2, cm^{-1}): ν_{NN} 2067.

UV-vis: See Table S1.

[Fe(depe)$_2$(σ-H$_2$)]$^+$[BAr$^F{}_4$]$^-$, [1·H$_2$]$^+$[BAr$^F{}_4$]$^-$: In a Ar-filled glove-box at 1 atm. pressure, [**1**]$^+$[BAr$^F{}_4$]$^-$ (13.3 mg) was dissolved in THF solvent (20 ml), from which 1 ml (overall concentration 0.5 mM) was used to fill a J. Youngs-adapted quartz cuvette, which was subsequently sealed and removed from the glove-box. The cuvette was subsequently freeze-thaw-degassed three times with H_2 on a Schlenk-line manifold (1 atm. pressure), with the last backfill at RT, before being subjected to UV-vis measurements. For NMR experiments solutions of [**1**]$^+$[BAr$^F{}_4$]$^-$ in DFB (0.4 ml, 1 mM concentration) were prepared under Ar in a J. Youngs NMR tube, which were subsequently freeze-thaw-degassed three times with H_2 on a Schlenk-line manifold. The last backfill of H_2 (1 atm.) was subsequently sealed at liquid N_2 temperature, before being warmed to RT (internal pressure ~ 4 atm.). Solution ESR experiments were treated analogously to NMR experiments, except that the last backfill was at RT and hence the internal pressure was 1 atm..

^{1}H NMR (400.4 MHz, DFB, 4 atm.H_2, 293 K) δ: –1.93 (br s).

UV-vis (DFB, 1 atm.H_2, –15°), nm {m^2 mol^{-1}}: 345 {60}, 850 {20}.

Preparation of samples for powder ESR: For powder ESR experiments, a stock solution of [**1**]$^+$[BAr$^F{}_4$]$^-$ in DFB (25 mM) was prepared in an Ar-filled glovebox. For each experiment, 0.2 mL of stock solution (corresponding to 0.005 mmol [**1**]$^+$[BAr$^F{}_4$]$^-$) was transferred to a J. Youngs-adapted quartz ESR tube. The solvent was carefully removed *in vacuo* to leave a thin powder coating of the sample on the walls of the tube, and the tube subsequently back-filled to a pressure of 1 atm. with Ar, N_2 or H_2.

Samples intended to contain precisely 1 eq. of N_2 or H_2 were initially prepared under Ar in same manner. The Ar was subsequently removed under vacuum, and 0.005 mmol of the appropriate gas was added through use of a Toepler pump.

***trans*-[Fe(depe)$_2$(H)(N$_2$)]$^+$[BAr$^F{}_4$]$^-$:** *trans*-Fe(depe)$_2$(H)(Cl) (250 mg, 0.495 mmol) and NaBAr$^F{}_4$ (461 mg, 0.520 mmol) were dissolved in 10 mL of Et_2O under a N_2 atmosphere and stirred for 12 h at RT. After filtration, the solution was concentrated *in vacuo* and then layered with pentane. Slow diffusion of pentane into the Et_2O solution yielded off-white crystals which were collected by filtration, washed with further pentane, and dried *in vacuo* (598 mg, 89%).

^{31}P {^{1}H} NMR (162 MHz, Et_2O) δ: 81.4 (s).

^{1}H NMR (400.4 Hz, Et_2O, hydride region) δ: – 18.3 (quintet, $^{2}J_{PH}$ = 48.9 Hz, Fe-H).

IR (Et_2O, cm^{-1}): ν_{NN} 2102

***trans*-[Fe(depe)$_2$(H)(H_2)]$^+$[BAr$^F{}_4$]$^-$:** *trans*-Fe(depe)$_2$(H)(Cl) (250 mg, 0.495 mmol) and NaBAr$^F{}_4$ (461 mg, 0.520 mmol) were weighed into a Schlenk flask in an Ar glove-box, before being sealed and transferred to a Schlenk-line manifold. H_2-saturated Et_2O (10 ml) was subsequently added under a H_2 atmosphere, the flask sealed, and then followed by stirring for 12 h at RT. The remaining operations were performed under Ar. After filtration, the solution was concentrated *in vacuo* and then layered with pentane. Slow diffusion of pentane into the Et_2O solution yielded off-white crystals which were collected by filtration, washed with further pentane, and dried *in vacuo* (534 mg, 81%).

^{31}P {^{1}H} NMR (162 MHz, Et_2O) *δ*: 93.4 (s).

^{1}H NMR (400.4 Hz, Et_2O, 298 K, hydride region) *δ*: – 11.0 (br multiplet, Fe-H).

2. NMR spectroscopy

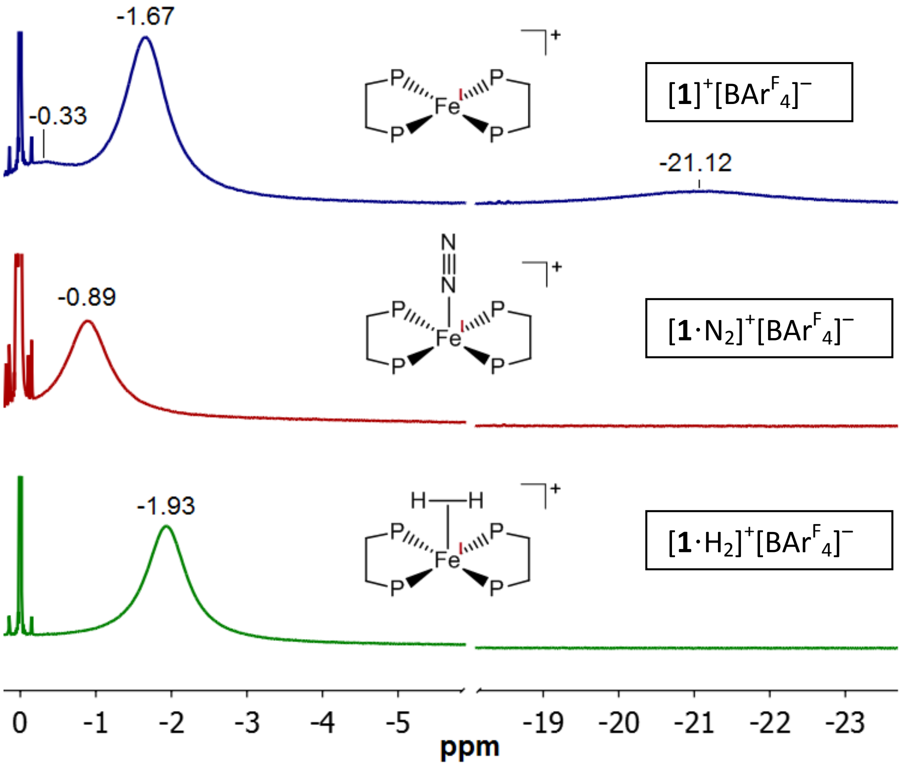

Figure S1. High field region of the ^{1}H NMR spectra of [**1**]$^{+}$[BAr$^{F}{}_{4}$]$^{-}$ in DFB under Ar (1 atm., blue), N_2 (4 atm., red), and H_2 (4 atm., green); referenced to $SiMe_4$ (δ = 0) in a capillary insert. P = PEt_2.

3. ESR spectroscopy

3.1 X-band CW data

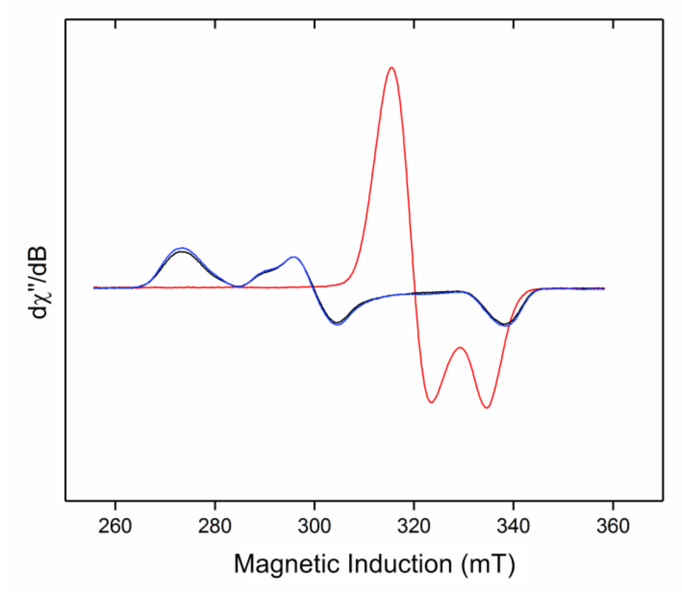

Figure S2. Reversible binding of N_2 by [**1**]$^+$[BAr$^F_4$]$^-$ in the solid-state: CW X-band ESR spectra of powdered [**1**]$^+$[BAr$^F_4$]$^-$ under Ar (black), followed by evacuation and subsequent admission of H_2 (1 atm., [**1**·N_2]$^+$[BAr$^F_4$]$^-$, pink), and sequential evacuation to 10^{-3} mbar and backfill with Ar (blue). All spectra recorded at 40 K.

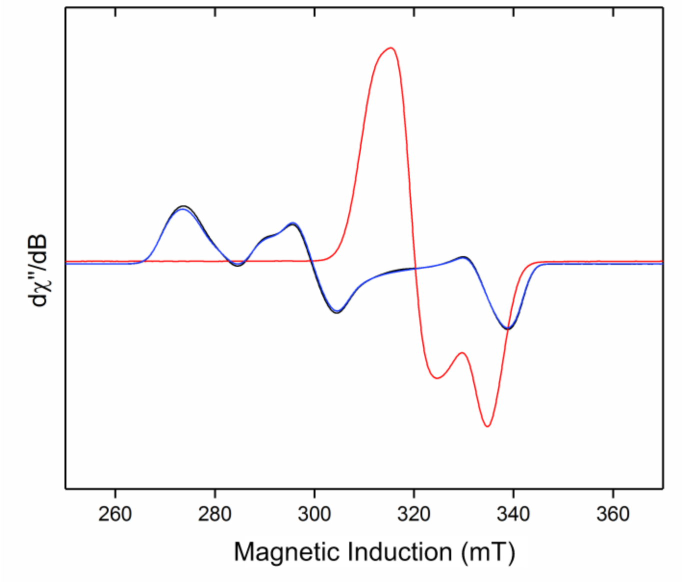

Figure S3. Reversible binding of H_2 by [**1**]$^+$[BAr$^F_4$]$^-$ in the solid-state: CW X-band ESR spectra of powdered [**1**]$^+$[BAr$^F_4$]$^-$ under Ar (black), followed by evacuation and subsequent admission of H_2 (1 atm., [**1**·H_2]$^+$[BAr$^F_4$]$^-$, pink), and sequential evacuation to 10^{-3} mbar and backfill with Ar (blue). All spectra recorded at 40 K.

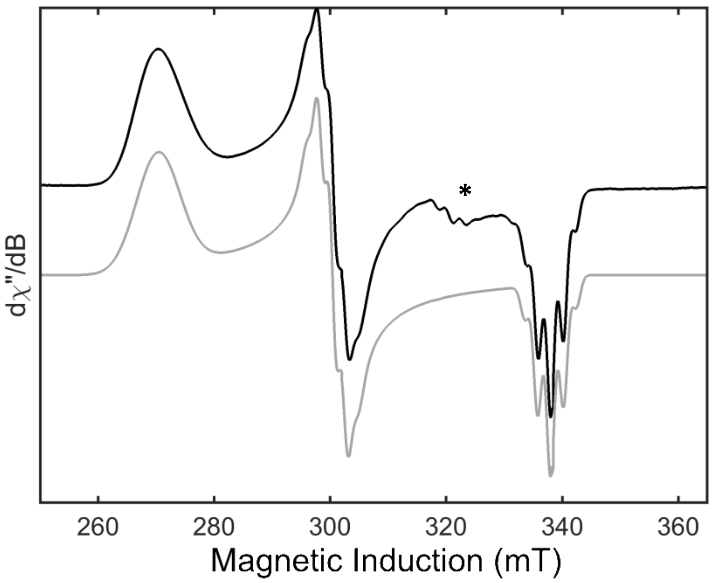

Figure S4. CW X-band ESR spectra of [**1**]$^{+}$[BAr$^{F}{}_{4}$]$^{-}$ at 2.5 mM in 7:1 PhMe:DFB under Ar, recorded as a frozen glass at 40 K and 50 μW, with a microwave frequency of 9.3905 GHz; black trace: experiment; grey trace: simulation. (*) corresponds to trace [**1·**(**N$_2$**)]$^{+}$[BAr$^{F}{}_{4}$]$^{-}$ impurity, due to N_2 contaminant in the Ar feed. Simulation parameters: $g_1 = 2.483$, $g_2 = 2.234$, $g_3 = 1.985$ with four ^{31}P hyperfine interactions of type 1, $\boldsymbol{A}(^{31}\text{P})$ = [66.6 63.2 62.6] /MHz and type 2, $\boldsymbol{A}(^{31}\text{P})$ = [69.9 59.5 61.8] /MHz, as collinear with g-tensor; isotropic linewidth of 0.3 /mT and anisotropic linewidth of $l_1 = 9.22$, $l_2 = 2.2$ and $l_3 = 1.6$ /mT.

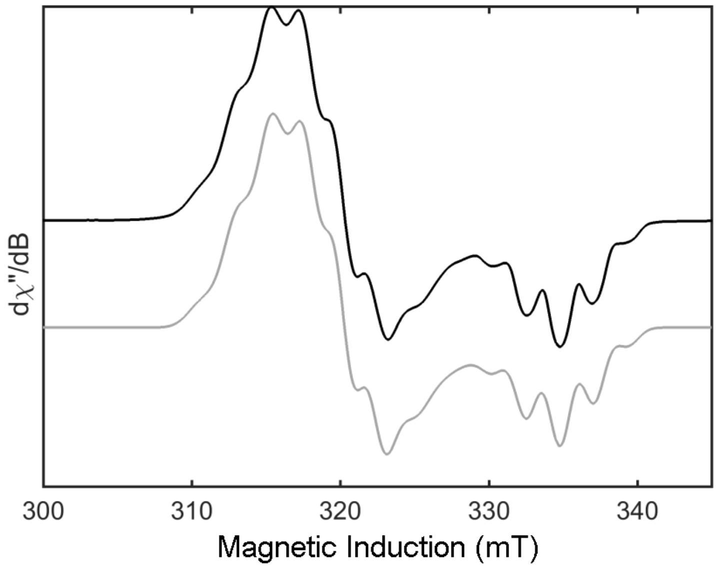

Figure S5. CW X-band ESR spectra of [**1·N$_2$**]$^{+}$[BAr$^{F}{}_{4}$]$^{-}$ at 2.5 mM in 7:1 PhMe:DFB under N_2, recorded as a frozen glass at 40 K and 50 μW, microwave power with a microwave frequency 9.3795 GHz; black trace: experiment; grey trace: simulation. Simulation parameters: $g_1 = 2.125$, $g_2 = 2.093$, $g_3 = 2.0016$ with four ^{31}P hyperfine interactions, two of type 1, $\boldsymbol{A}(^{31}\text{P})$ = [65.6 61.4 60.9] /MHz and two of type 2, $\boldsymbol{A}(^{31}\text{P})$ = [72 61.7 64.3] /MHz, as collinear with g-tensor; isotropic linewidth of 0.3 /mT and anisotropic linewidth of $l_1 = 2.4$, $l_2 = 2$ and $l_3 = 1.7$ /mT.

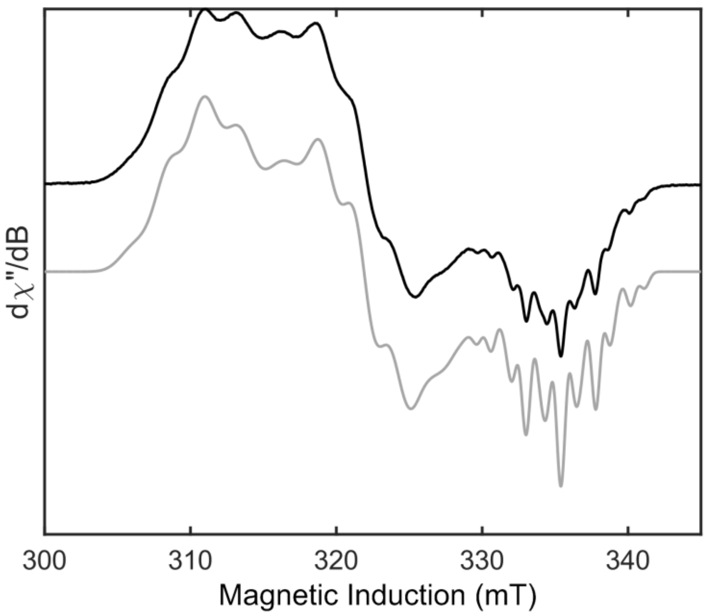

Figure S6. CW X-band ESR spectra of [**1**·**H_2**]$^+$[$BAr^F{}_4$]$^-$ at 2.5 mM in 7:1 PhMe:DFB under H_2, recorded as a frozen glass at 40 K and 50 μW, microwave power with a microwave frequency 9.3795 GHz; black trace: experiment; grey trace: simulation. Simulation parameters: $g_1 = 2.158$, $g_2 = 2.084$, $g_3 = 2.00025$ with four ^{31}P hyperfine interactions, $\boldsymbol{A}(^{31}P)$ = [66.8 66.5 65.5; 66.8 66.5 65.5; 70.2 69.4 68.4; 70.2 69.4 68.4] /MHz, with corresponding Euler angles (α,β,γ): [149 1 -149; -96 11 -84; 98 85 -175; 98 80 4] /deg. Hyperfine values for ^1H are $\boldsymbol{A}(^1H)$ = [-17.99 -19.93 26.58] /MHz; isotropic linewidth of 0.3 /mT and anisotropic linewidth of $l_1 = 2.4$, $l_2 = 2$ and $l_3 = 1.7$ /mT.

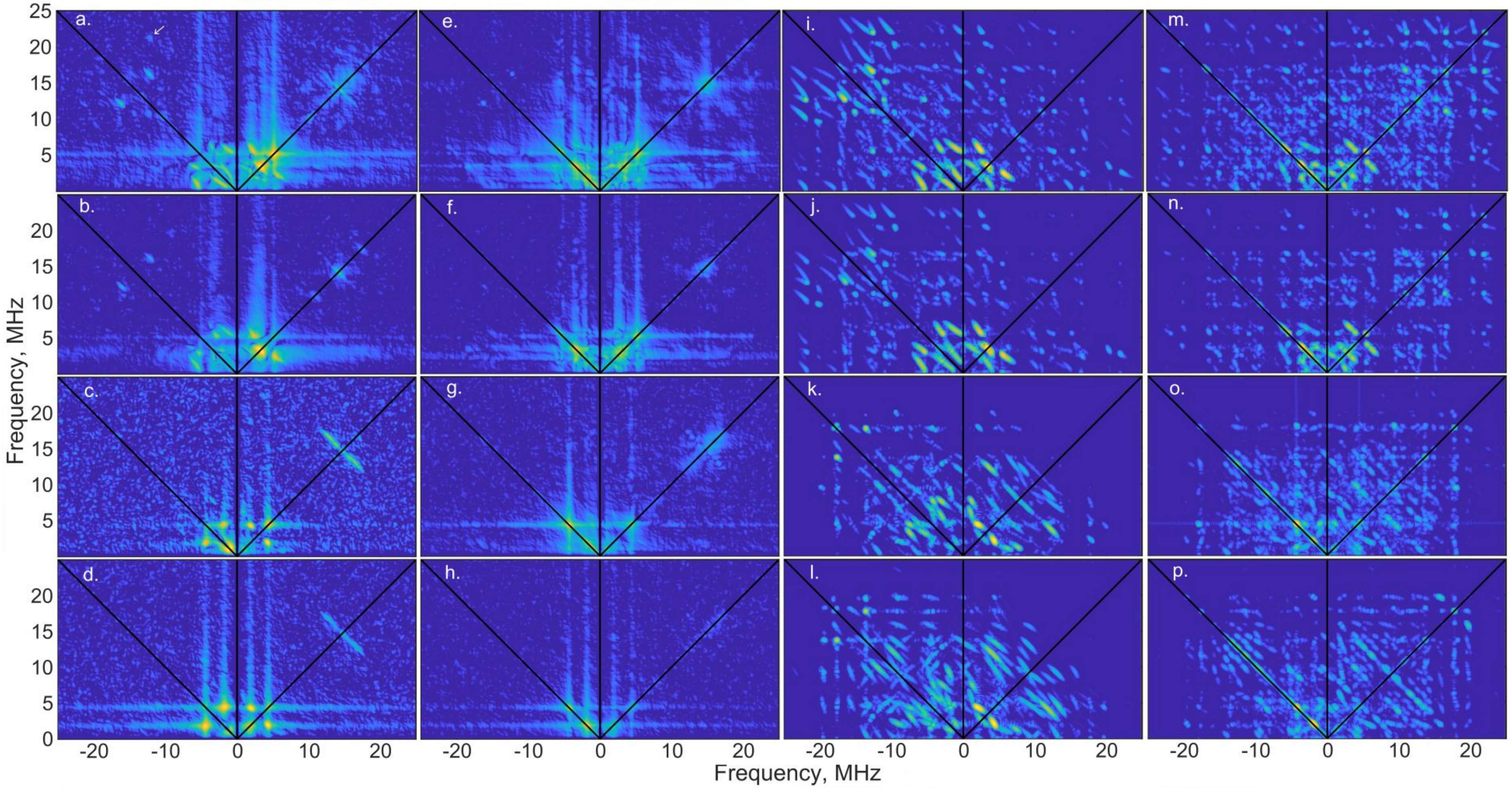

Figure S7. Intensity map plots of standard 4-pulse HYSCORE (a-d, i-l) and DONUT-HYSCORE(e-h, m-p) in data (a-h) and simultaneous simulation of two ^{14}N nuclei (i-p). Acquisition parameters and simulations parameters are in the main text and fig. 3.

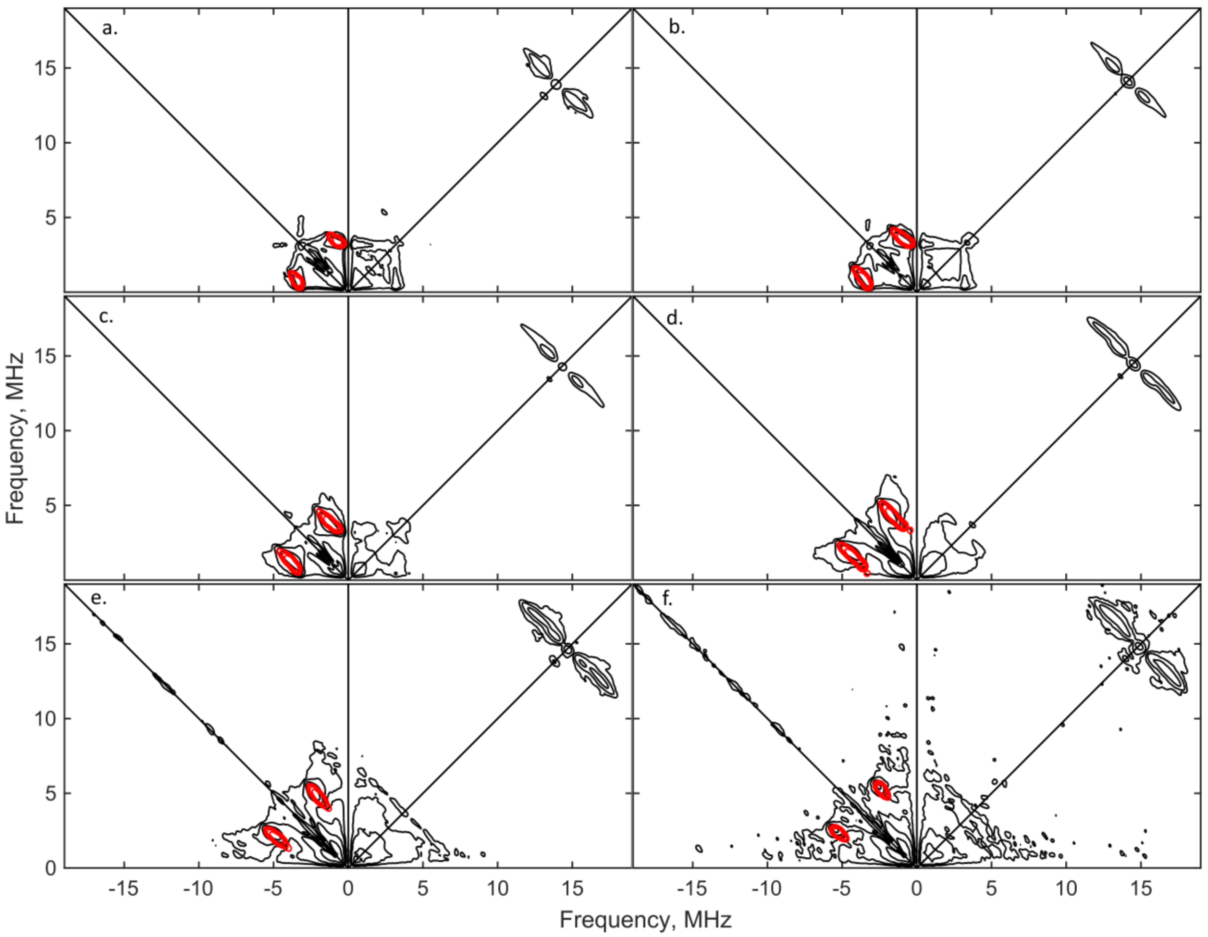

Figure S8. Field-dependent ESR simulation (red) for the 4-pulse HYSCORE signal (black) of a [**1**·(^{15}**N$_2$**)]$^+$[BAr$^F{}_4$]$^-$, at [field (G), g-value] a. 3261, 2.134; b. 3304, 2.106; c. 3348, 2.070; d. 3392, 2.052; e. 3436, 2.025; f. 3480, 1.999, with simulation values of $\boldsymbol{A}$(^{15}N) = [3.5 6.7 8.3] MHz, A_{iso}(^{15}N) = 6.2 MHz. The microwave frequency was 9.7615 GHz, with $t_1 = t_2$ = 100 ns, τ = 200 ns, and a time step of 20 ns. Microwave pulse lengths were $\pi/2$ = 8 ns and π = 12 ns.

4. IR spectroscopy

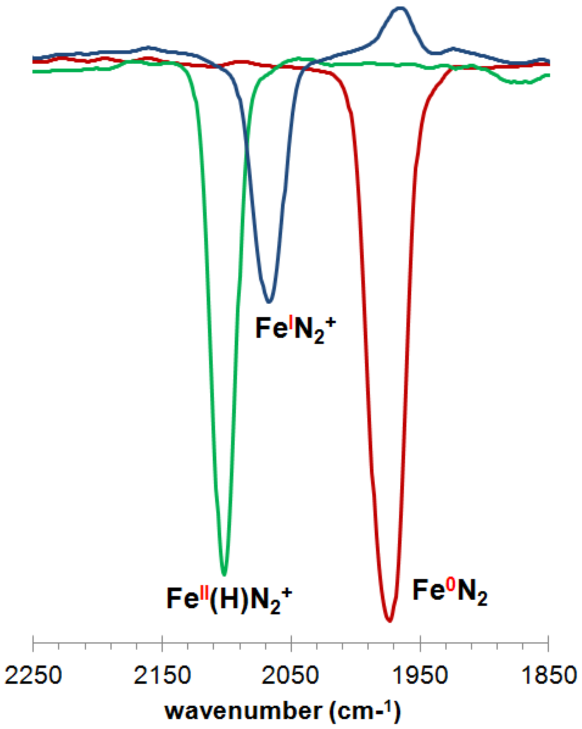

Figure S9. IR spectra of the ν_{NN} region: **1**·N_2 (red), [**1**·N_2]$^+$[BAr$^F{}_4$]$^-$ (from [**1**]$^+$[BAr$^F{}_4$]$^-$ in a glove-box under a N_2 atmosphere; blue), and *trans*-[Fe(depe)$_2$(H)(N_2)]$^+$[BAr$^F{}_4$]$^-$ (green). All prepared as 0.1 M Et_2O solutions.

5. UV-vis spectroscopy

General considerations

Notably, variable temperature UV-Vis spectra of **[1]$^+$[BAr$^F_4$]$^-$** in DFB of THF solutions are virtually identical, and both are essentially invariant over the range 298-343 K, which was used for the N_2 binding studies. This strongly implies that [**1**]$^+$ does not coordinate these solvents, despite their significantly different donor properties.

Table S1 Electronic absorption (UV-vis) features of **[1]$^+$** and **[1**·N_2**]$^+$**, and Fe(I) complexes reported by Tyler *et al.*.[12]

Complex	λ_{max}/nm (ε_{max}/M cm^{-1})	Ref.
**[1]$^+$ [BAr$^F_4$]$^-$** (DFB, 298 K, Ar)	378 (~103)^a<br>414 (~94)^a<br>496 (121)<br>618 (285)	this work
**[1·N_2]$^+$ [BAr$^F_4$]$^-$** (DFB, –15°C, N_2)	359 (316)<br>1018 (55)	this work
**{[Fe(DMeOPrPE)$_2$]$_2$(μ-N_2)}[BPh$_4$]$_2$** (THF)^b	~350^c<br>480<br>580	12
**[Fe(N_2)(DMeOPrPE)$_2$][BPh$_4$]** (THF)^b	~360^c<br>1010	12

^{a} overlapping bands; ^{b} ε_{max} values not reported; ^{c}extracted visually from ref. [12].

DMeOPrPE = [MeOCH$_2$CH$_2$CH$_2$)$_2$PCH$_2$]$_2$.

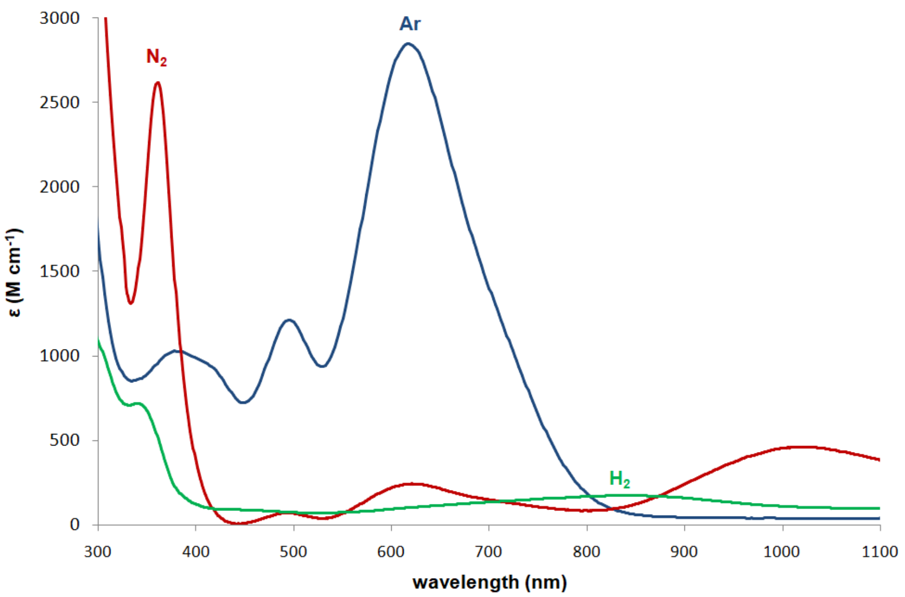

Figure S10. UV-vis spectra of [**1**]$^+$[BAr$^F_4$]$^-$ under Ar (blue), N_2 (red), and H_2 (green) atmosphere in DFB (293 K, 1 atm.).

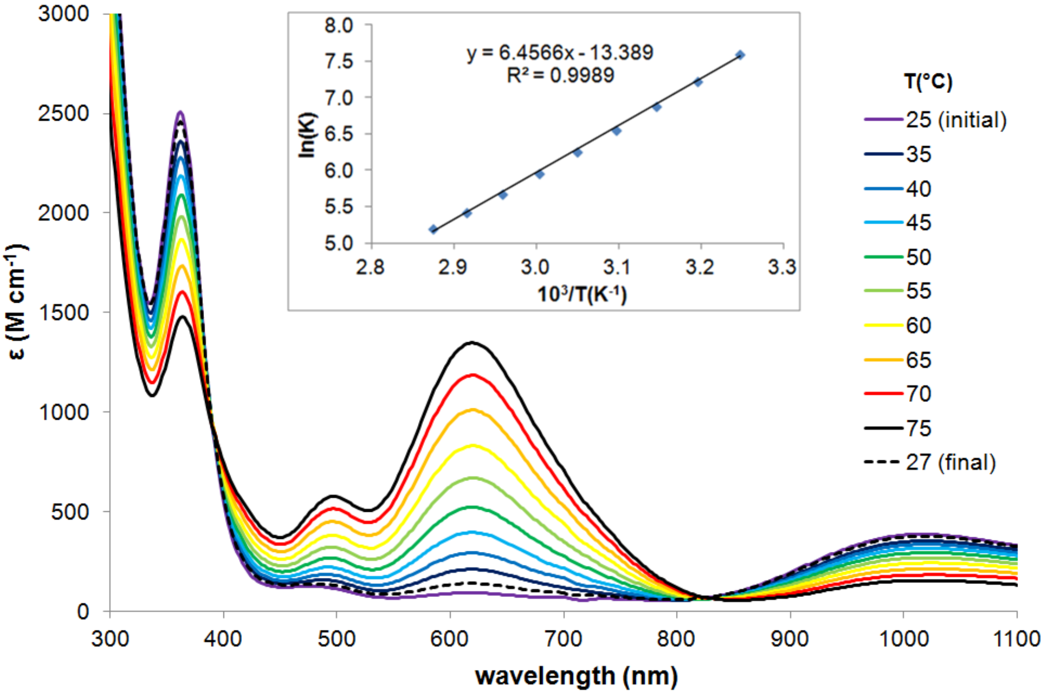

Figure S11. Variable-temperature UV-vis spectra of [**1**]$^+$[BAr$^F_4$]$^-$ and [**1**·N_2]$^+$[BAr$^F_4$]$^-$ in THF under 1 atm. N_2; van't Hoff plot (inset) derived from changes in the absorbance at 618 nm between 35 and 75 °C; the concentration of N_2 in THF was taken to be 6.4 mM using solubility data from ref. [8]. The presence of clearly defined isosbestic points corroborates a completely reversible equilibrium solely involving two Fe-containing complexes and N_2.

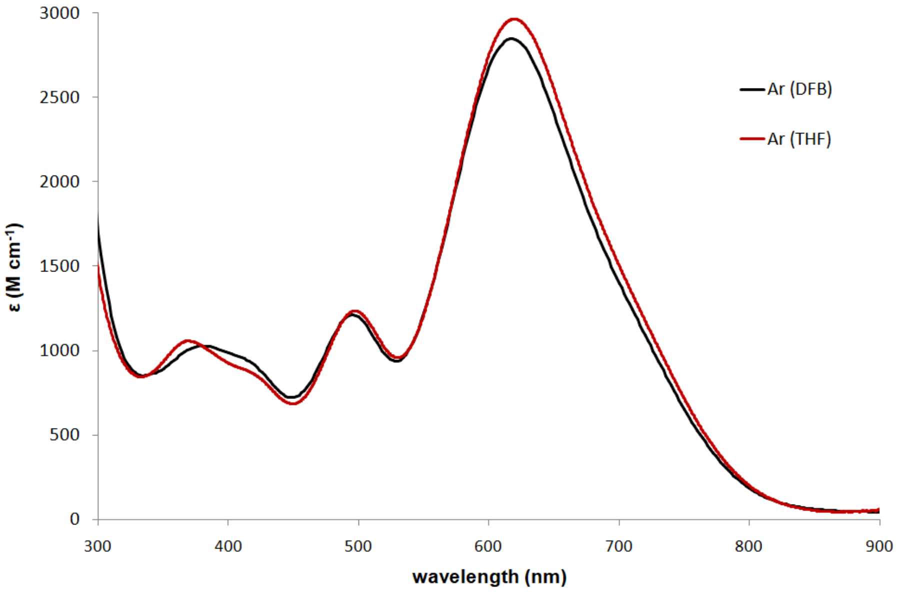

Figure S12. UV-vis spectra of [**1**]$^{+}$[BAr$^{F}_{4}$]$^{-}$ in DFB and THF (293 K, 1 atm Ar).

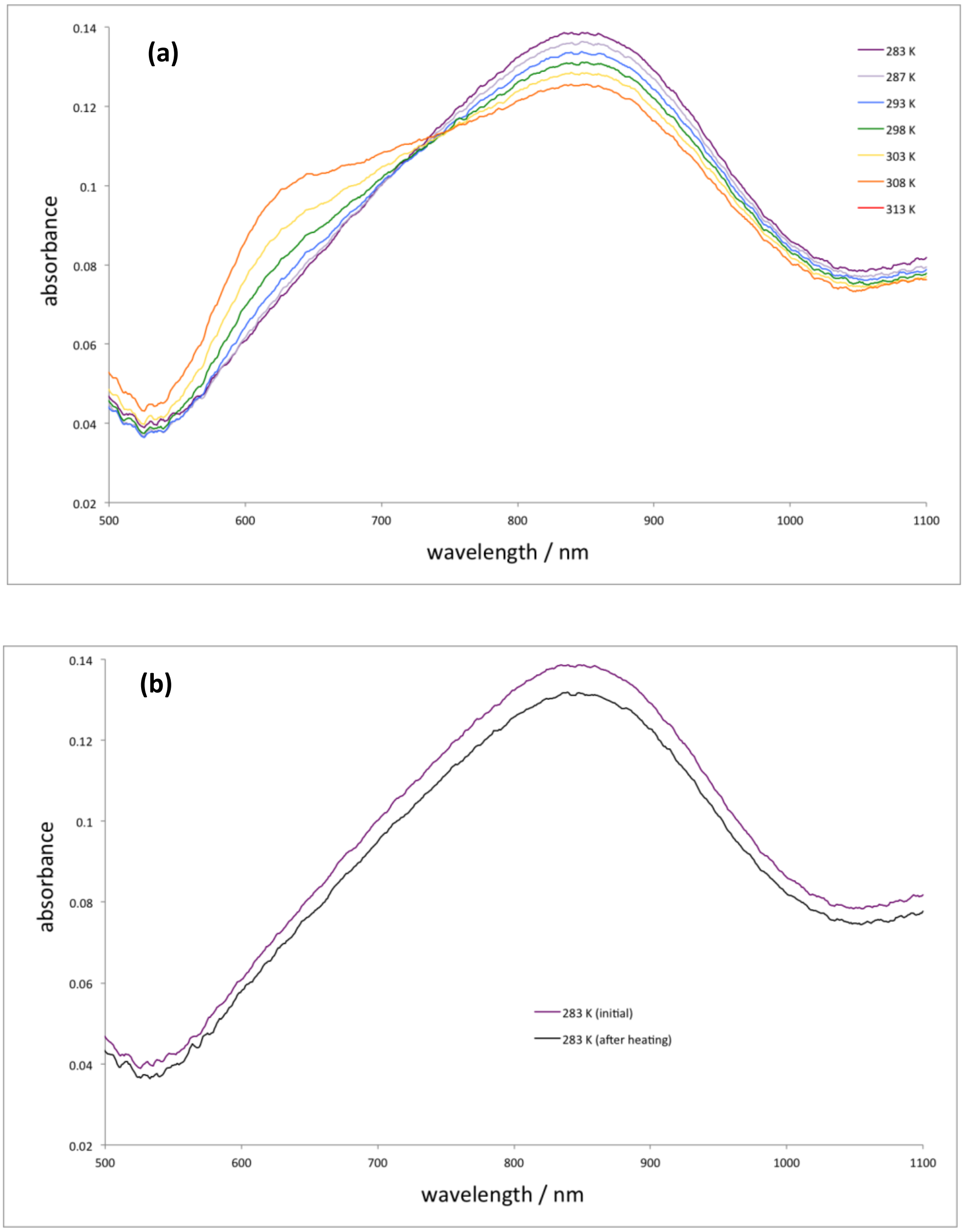

Figure S13. (a) Variable temperature UV-Vis spectra of [**1**]$^+$[BAr$^F{}_4$]$^-$ taken under an atmosphere of H_2 (1 atm.) are consistent with reversible release of H_2 from [**1**·H_2]$^+$[BAr$^F{}_4$]$^-$ to form [**1**]$^+$[BAr$^F{}_4$]$^-$ (evidenced by the increasing absorption centred at 618 nm), as temperature is increased. **(b)** Partial decomposition at elevated temperature is evidenced by reduced absorption upon returning to low temperature.

6. Computational data

Density functional calculations were carried out using the ADF program suite version 2014.1.[13] The Slater-type orbital (STO) basis sets were of triple-**ζ** quality augmented with a one polarization function (ADF basis TZP). Core electrons were frozen (C, N 1s; Fe 2p) in our model of the electronic configuration for each atom. The local density approximation (LDA) by Vosko, Wilk and Nusair (VWN)[14] was used together with the exchange correlation corrections of Becke and Perdew (BP86).[15] Optimized geometries were ascertained as local mimima *via* frequency calculations. Time-dependent DFT (TDDFT) calculations for calculating electronic absorption spectra used a SAOP functional and a QZ4P all-electron basis set.

Table S2. Selected metric parameters for geometry optimised structures of $[Fe(depe)_2]^+$ ($[\mathbf{1}]^+$), σ-N_2 and σ-H_2 complexes thereof ($[\mathbf{1}\cdot N_2]^+$ and $[\mathbf{1}\cdot(H_2)]^+$), and oxidative addition product with H_2 ($[\mathbf{1}\cdot(H)_2]^+$).

		$[\mathbf{1}]^+$	$[\mathbf{1}\cdot N_2]^+$ $[OTf]^{-a}$	$[\mathbf{1}\cdot N_2]^+$	$[\mathbf{1}\cdot(H_2)]^+$	$[\mathbf{1}\cdot(H)_2]^+$
**Distances (Å)**	**Fe-P**	2.250	2.2471(9)<br>2.2512(9)	2.274<br>2.293	2.256	2.217<br>2.225<br>2.280<br>2.293
	**Fe-H**	-	-	-	1.613<br>1.615	1.507<br>1.511
	**Fe-N**	-	1.857(6)	1.831	-	-
	**N-N**	-	1.121(9)	1.135	-	-
	**H···H**	-	-	-	0.899	1.567
**Angles (°)**	**P-Fe-P**	85.28<br>94.91	83.83(3)<br>95.03(3)	83.87<br>94.71	85.53<br>85.52<br>94.06<br>94.08	85.33<br>86.50<br>101.54<br>103.07
	**H-Fe-H**	-	-	-	32.35	62.55
	**Dihedral between depe ligand PP planes**	6.90	15.39	18.86	16.92	64.05

[a] From X-ray crystallographic data in reference [1]. See also CCDC 1451414.

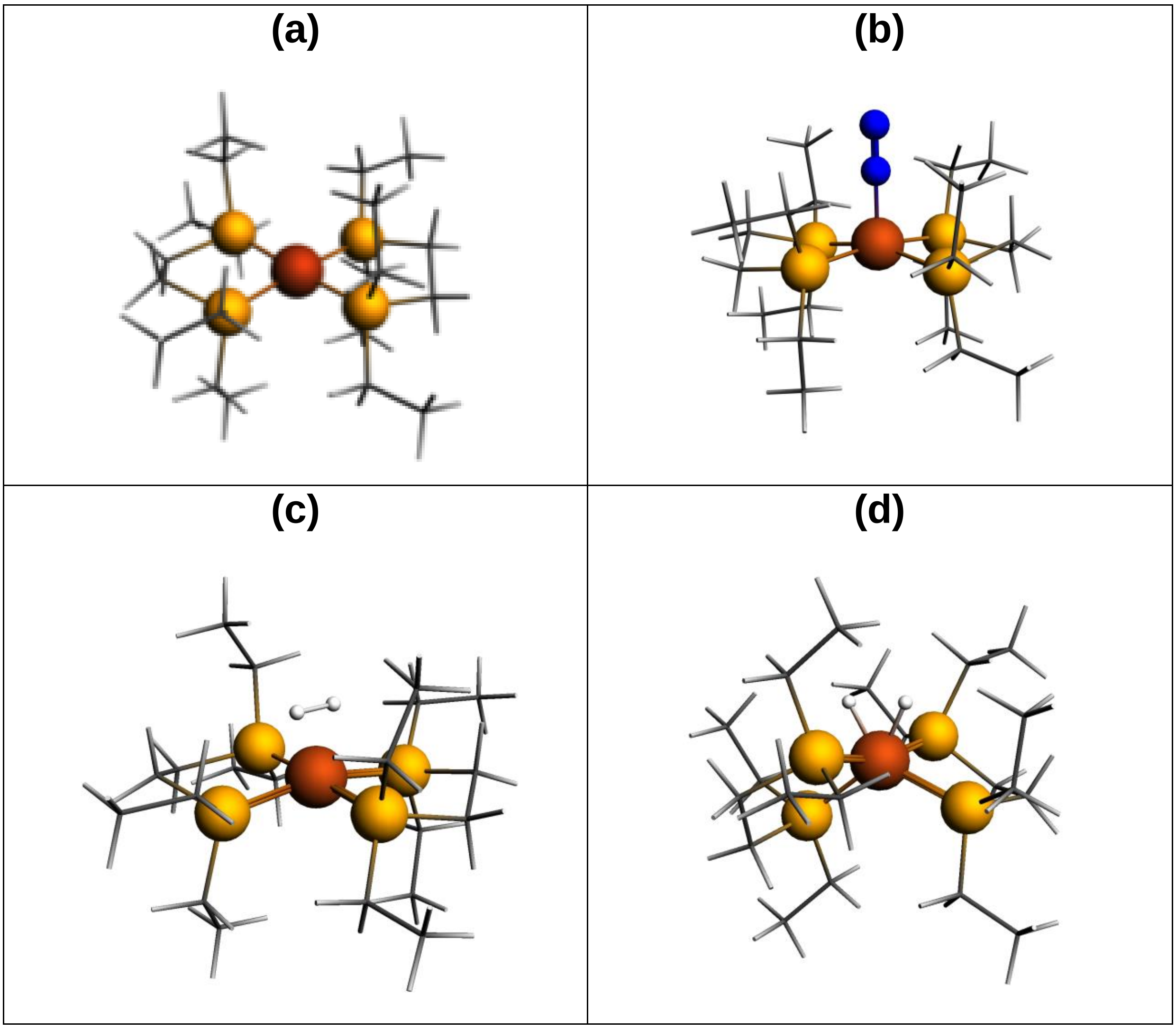

Figure S14. DFT-optimised structures of (a) [**1**]$^+$, (b) [**1**·N_2]$^+$, (c) [**1**·H_2]$^+$, (d) [**1**$(H)_2$]$^+$.

Table S3. Calculated low-energy of absorptions of [**1**]$^+$, [**1**·$\mathbf{N_2}$]$^+$ and [**1**·$\mathbf{H_2}$]$^+$.

Transition	Wavelength [**1**]$^+$	Oscillator strength [**1**]$^+$	Transition	Wavelength [**1**·$\mathbf{N_2}$]$^+$ [**1**·$\mathbf{H_2}$]$^+$	Oscillator strength [**1**·$\mathbf{N_2}$]$^+$ [**1**·$\mathbf{H_2}$]$^+$
xz > z^2	825	0.000	xz/yz > z^2	660 677	0.004 0.006
yz > z^2	758	0.000	yz/xz > z^2	629 565	0.006 0.010
xz > z	587	0.011	x^2-y^2 > z^2	574 606	0.003 0.000
yz > z	548	0.015			
x^2-y^2 > z	503	0.001			

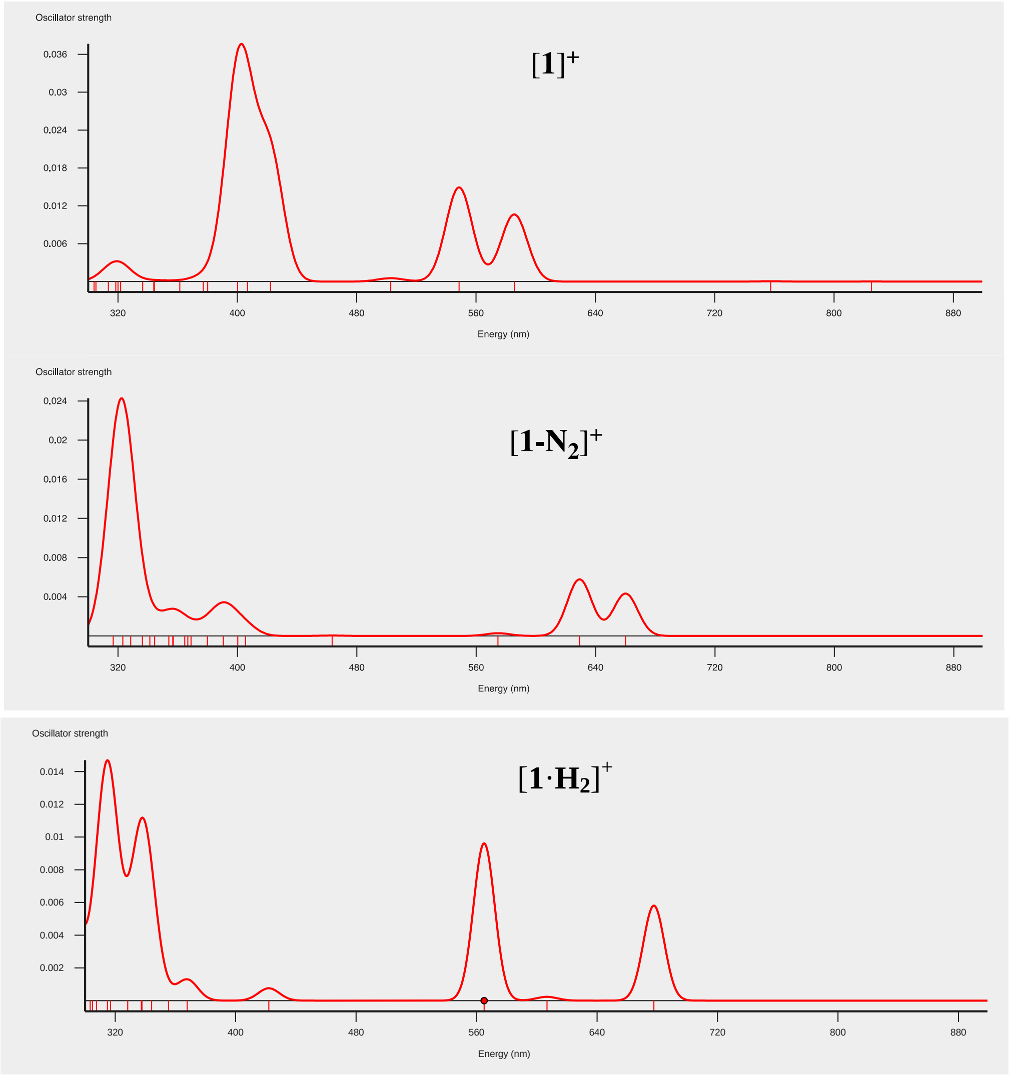

Figure S15. Calculated absorption spectra for [**1**]$^+$, [**1**·**N**$_2$]$^+$ and [**1**·**H**$_2$]$^+$.

Cartesian coordinates for optimised geometries

$[Fe(depe)_2]^+$; $[1]^+$

73

Fe	0.00000000	-0.00000000	0.00000000
P	-1.52472518	-1.65627104	0.07927117
C	-0.72042791	-3.32483472	-0.25934464
H	-0.73827907	-3.46700295	-1.35226120
H	-1.31695348	-4.13654377	0.18404587
C	0.72042791	-3.32483472	0.25934464
H	1.31695348	-4.13654377	-0.18404587
H	0.73827907	-3.46700295	1.35226120
P	1.52472518	-1.65627104	-0.07927117
C	-2.37049256	-1.91280624	1.73442709
H	-2.82159134	-2.91752161	1.73415266
H	-3.20136109	-1.19099454	1.77140557
C	-1.45952947	-1.72375859	2.95458485
H	-2.03661439	-1.81090966	3.88647160
H	-0.66184554	-2.47935500	2.99362449
H	-0.98401634	-0.73075601	2.94668085
C	-2.97927866	-1.69155628	-1.10071159
H	-2.55554339	-1.51330543	-2.10267705
H	-3.58749276	-0.80414601	-0.86421549
C	-3.86333948	-2.94561278	-1.10978220
H	-4.66932449	-2.83150937	-1.84859996
H	-3.29713465	-3.84683216	-1.38144451
H	-4.33692528	-3.12244171	-0.13443564
C	2.97927866	-1.69155628	1.10071159
H	2.55554339	-1.51330543	2.10267705
H	3.58749276	-0.80414601	0.86421549
C	3.86333948	-2.94561278	1.10978220
H	4.66932449	-2.83150937	1.84859996
H	3.29713465	-3.84683216	1.38144451
H	4.33692528	-3.12244171	0.13443564
C	2.37049256	-1.91280624	-1.73442709
H	2.82159134	-2.91752161	-1.73415266
H	3.20136109	-1.19099454	-1.77140557
C	1.45952947	-1.72375859	-2.95458485
H	2.03661439	-1.81090966	-3.88647160
H	0.66184554	-2.47935500	-2.99362449
H	0.98401634	-0.73075601	-2.94668085
P	1.52472518	1.65627104	0.07927117
C	0.72042791	3.32483472	-0.25934464
H	0.73827907	3.46700295	-1.35226120
H	1.31695348	4.13654377	0.18404587
C	-0.72042791	3.32483472	0.25934464
H	-1.31695348	4.13654377	-0.18404587
H	-0.73827907	3.46700295	1.35226120
P	-1.52472518	1.65627104	-0.07927117
C	2.37049256	1.91280624	1.73442709
H	2.82159134	2.91752161	1.73415266
H	3.20136109	1.19099454	1.77140557
C	1.45952947	1.72375859	2.95458485
H	2.03661439	1.81090966	3.88647160
H	0.66184554	2.47935500	2.99362449

H	0.98401634	0.73075601	2.94668085
C	2.97927866	1.69155628	-1.10071159
H	2.55554339	1.51330543	-2.10267705
H	3.58749276	0.80414601	-0.86421549
C	3.86333948	2.94561278	-1.10978220
H	4.66932449	2.83150937	-1.84859996
H	3.29713465	3.84683216	-1.38144451
H	4.33692528	3.12244171	-0.13443564
C	-2.97927866	1.69155628	1.10071159
H	-2.55554339	1.51330543	2.10267705
H	-3.58749276	0.80414601	0.86421549
C	-3.86333948	2.94561278	1.10978220
H	-4.66932449	2.83150937	1.84859996
H	-3.29713465	3.84683216	1.38144451
H	-4.33692528	3.12244171	0.13443564
C	-2.37049256	1.91280624	-1.73442709
H	-2.82159134	2.91752161	-1.73415266
H	-3.20136109	1.19099454	-1.77140557
C	-1.45952947	1.72375859	-2.95458485
H	-2.03661439	1.81090966	-3.88647160
H	-0.66184554	2.47935500	-2.99362449
H	-0.98401634	0.73075601	-2.94668085

$[Fe(depe)_2(\sigma\text{-}N_2)]^+$; $[1\cdot N_2]^+$

75

Fe	3.88294410	6.00660040	8.49846094
P	5.24070277	6.34593709	6.70645994
C	6.96988254	5.81784151	7.17809495
H	6.96436148	4.71722990	7.22638468
H	7.69824512	6.10962708	6.40567594
C	7.29989159	6.42061333	8.54623532
H	7.49060522	7.50123546	8.45482778
H	8.19853008	5.97009723	8.99364602
P	5.82794933	6.19715634	9.69743544
N	3.88294163	4.17597878	8.49846255
N	3.88294010	3.04118176	8.49846355
C	5.43375801	8.15646771	6.26001026
H	4.47817017	8.44269180	5.79311595
H	5.47388957	8.70268046	7.21581237
C	6.60901464	8.56238924	5.36097779
H	6.58271735	8.05829181	4.38511895
H	7.58046469	8.34687772	5.82776115
H	6.57388113	9.64427686	5.16772938
C	5.01485614	5.56110882	5.02324307
H	4.00432755	5.83577503	4.67970036
H	5.72039078	6.05736282	4.33815577
C	5.20911772	4.03977013	4.96308804
H	4.97641770	3.67012190	3.95463801
H	4.56185092	3.50747151	5.67340344
H	6.24771873	3.75564955	5.18029199
C	6.14286119	7.52156954	10.98104848
H	5.41922366	7.35336695	11.79524611
H	7.13959103	7.33137331	11.40811862
C	6.05563573	8.96528317	10.47108139
H	6.86463167	9.19622782	9.76406773
H	6.14886042	9.67259768	11.30722331

H	5.09937735	9.16650306	9.96648983
C	6.25998497	4.62570365	10.61758247
H	5.37644256	4.37160047	11.22282072
H	6.31823862	3.84664229	9.84139277
C	7.52353505	4.61403747	11.48603979
H	8.42343094	4.90102999	10.92319596
H	7.69603458	3.59977808	11.87375220
H	7.43744030	5.28329943	12.35266696
P	2.52518634	6.34594391	10.29046133
C	0.79600515	5.81785216	9.81882725
H	0.80152324	4.71724046	9.77053947
H	0.06764335	6.10964106	10.59124575
C	0.46599773	6.42062246	8.45068582
H	0.27528701	7.50124527	8.54209145
H	-0.43264198	5.97010800	8.00327591
P	1.93793938	6.19715947	7.29948609
C	2.33213598	8.15647585	10.73690782
H	3.28772459	8.44269818	11.20380163
H	2.29200589	8.70268702	9.78110474
C	1.15688045	8.56240213	11.63593957
H	1.18317638	8.05830635	12.61179930
H	0.18542982	8.34689241	11.16915659
H	1.19201687	9.64428999	11.82918607
C	2.75103085	5.56111801	11.97367959
H	3.76156018	5.83578210	12.31722181
H	2.04549755	6.05737512	12.65876601
C	2.55676516	4.03977995	12.03383731
H	2.78946420	3.67013287	13.04228799
H	3.20403053	3.50747833	11.32352285
H	1.51816340	3.75566178	11.81663386
C	1.62303109	7.52157125	6.01587072
H	2.34666817	7.35336527	5.20167339
H	0.62630074	7.33137695	5.58880092
C	1.71026044	8.96528555	6.52583526
H	0.90126513	9.19623363	7.23284851
H	1.61703766	9.67259883	5.68969209
H	2.66651937	9.16650375	7.03042646
C	1.50589950	4.62570632	6.37934184
H	2.38944122	4.37159969	5.77410404
H	1.44764375	3.84664649	7.15553292
C	0.24234939	4.61404202	5.51088454
H	-0.65754573	4.90103795	6.07372787
H	0.06984712	3.59978241	5.12317392
H	0.32844594	5.28330221	4.64425619

$[Fe(depe)_2(\sigma\text{-}H_2)]^+$; $[1\cdot H_2]^+$

75

**Fe**	**0.00093938**	**-0.00075569**	**-0.21827765**
**P**	**1.69813783**	**1.44840069**	**0.11146990**
**C**	**1.11993578**	**3.18053885**	**-0.30202042**
**H**	**1.19926761**	**3.27524907**	**-1.39562383**
**H**	**1.79098411**	**3.92883234**	**0.14360150**
**C**	**-0.32457845**	**3.37580253**	**0.15390476**
**H**	**-0.79445675**	**4.24100535**	**-0.33607020**

H	-0.36313628	3.55918181	1.23732526
P	-1.33010637	1.82051639	-0.16476464
C	2.41799879	1.63035026	1.83005407
H	3.08855715	2.50256594	1.82062212
H	3.05280665	0.74626095	1.98959825
C	1.39777012	1.75128080	2.96640992
H	1.90648276	1.73302422	3.93995498
H	0.83335723	2.69148552	2.91421604
H	0.67670651	0.92178261	2.94679819
C	3.24269062	1.31226224	-0.93148901
H	2.88319147	1.20380452	-1.96678315
H	3.71600502	0.35334936	-0.67160721
C	4.26951964	2.44812784	-0.83755284
H	5.11168724	2.24541060	-1.51367833
H	3.84108359	3.41472539	-1.13217228
H	4.68258307	2.55166739	0.17415146
C	-2.66837127	1.97671907	1.13522639
H	-2.18430214	1.69393724	2.08304564
H	-3.40521926	1.18774563	0.92189918
C	-3.37794149	3.32886196	1.28109765
H	-4.10565629	3.28062896	2.10319844
H	-2.67687574	4.14092593	1.51420449
H	-3.93140980	3.60832034	0.37544966
C	-2.26719454	2.25542063	-1.72598157
H	-2.91744033	3.10989621	-1.48445957
H	-2.92835997	1.40428890	-1.94471256
C	-1.40736019	2.58750886	-2.94932513
H	-2.05027109	2.80104862	-3.81401166
H	-0.78631228	3.47824566	-2.78138633
H	-0.74547077	1.75635808	-3.22585301
P	-1.69586620	-1.44942364	0.11309865
C	-1.11828154	-3.18255029	-0.29743797
H	-1.20001882	-3.28013488	-1.39059713
H	-1.78871110	-3.92958360	0.15127424
C	0.32705714	-3.37714839	0.15617146
H	0.79666127	-4.24209389	-0.33452648
H	0.36719012	-3.56057132	1.23952327
P	1.33218912	-1.82133826	-0.16286709
C	-2.41889473	-1.62898061	1.83046934
H	-3.09509922	-2.49680542	1.81892644
H	-3.04799974	-0.74100818	1.99068675
C	-1.40100905	-1.75891318	2.96805233
H	-1.91090038	-1.73974494	3.94091656
H	-0.84263347	-2.70267876	2.91438557
H	-0.67458083	-0.93402288	2.95137695
C	-3.23891146	-1.31284487	-0.93203754
H	-2.87871828	-1.20336770	-1.96691378
H	-3.71218796	-0.35412377	-0.67127633
C	-4.26630799	-2.44840462	-0.84008542
H	-5.10739695	-2.24527177	-1.51742186
H	-3.83771320	-3.41513941	-1.13413682
H	-4.68100168	-2.55201240	0.17094287
C	2.66939290	-1.97640572	1.13855740
H	2.18380195	-1.69539489	2.08612837
H	3.40548715	-1.18626474	0.92707361
C	3.38116905	-3.32757799	1.28378104
H	4.10540773	-3.27993253	2.10898611
H	2.68112691	-4.14197407	1.51179182

H	3.93898208	-3.60315097	0.37962596
C	2.27094238	-2.25715019	-1.72275479
H	2.91448249	-3.11677619	-1.48167115
H	2.93841663	-1.40977089	-1.93682287
C	1.41164534	-2.57938142	-2.94904870
H	2.05479909	-2.80279986	-3.81108981
H	0.77795624	-3.46139639	-2.78243350
H	0.76208151	-1.73978156	-3.22887832
H	-0.35659601	-0.27498033	-1.76712898
H	0.35692294	0.27245771	-1.77003801

$[Fe(depe)_2(H)_2)]^+$; $[1(H)_2]^+$

75

Fe	15.95646613	9.64706536	2.86285803
P	15.16535851	11.62019253	3.68590408
P	14.24031053	9.75124905	1.46383989
P	17.40333240	8.99544781	4.42221374
P	17.73167849	9.63741242	1.41154558
H	18.43452309	8.64230971	7.41698719
H	19.77276693	9.36932320	6.50459993
C	14.61933547	11.89044730	5.44586175
H	15.53100026	11.97457891	6.05489112
H	14.12834678	12.87505032	5.47796652
C	14.30033611	9.22689566	-0.32434690
H	13.42644222	9.66534680	-0.83101927
H	15.18625858	9.71162958	-0.76292097
C	17.94400724	8.24467018	0.19061456
H	17.11706938	8.34581552	-0.52735312
H	18.87389792	8.43849076	-0.36470586
C	13.66967711	11.52631674	1.32583690
H	14.40549138	12.05036333	0.69642918
H	12.69648648	11.59971928	0.81917226
C	17.97175157	11.14472252	0.33118919
H	17.09680384	11.18507233	-0.33597888
H	17.87961797	12.00699137	1.00858742
C	13.62821059	12.12336454	2.73345968
H	12.76161477	11.73628756	3.28940677
H	13.53439724	13.21845346	2.71904368
H	14.97699667	8.99280848	3.80285963
C	16.33114921	13.05983514	3.39162069
H	17.29454292	12.76456559	3.83377754
H	16.48963284	13.08854375	2.30272967
C	17.96617201	6.83284966	0.78364832
H	17.96758980	6.08615508	-0.02200846
H	17.08933188	6.64806240	1.41959658
H	18.86794212	6.65971791	1.38585059
C	12.72427799	8.86386619	2.09449291
H	13.01611065	7.80482328	2.15907252
H	12.60011949	9.19204424	3.13685728
C	17.78299468	10.08285828	5.89749728
H	18.11770744	11.04729571	5.48316591
H	16.81103422	10.27711706	6.37426698
H	18.92083083	10.31386020	7.73122580
C	15.86026831	7.24616031	6.10383859
H	14.94814033	7.50577311	5.54984241

H	15.93593265	7.91422638	6.97313872
C	19.27265390	11.24400251	-0.47566095
H	19.29548018	12.19297438	-1.02912083
H	19.36579426	10.43633515	-1.21245714
H	20.15952071	11.22104929	0.17102254
C	13.68943070	10.81147427	6.00537603
H	12.74432033	10.75695113	5.44787085
H	13.43588863	11.03486369	7.05052749
H	14.15661383	9.81888682	5.97067370
C	14.34016024	7.71056352	-0.54412440
H	14.49900023	7.48009575	-1.60625616
H	13.39674587	7.23617877	-0.24488740
H	15.14541329	7.23415554	0.03059158
H	15.73899958	6.22395755	6.48667105
C	19.09649832	8.69874422	3.65899394
H	19.88847063	8.85422505	4.40466430
H	19.12572514	7.63605325	3.37966147
C	17.09455463	7.33386166	5.20469516
H	17.00281210	6.63216214	4.36227810
H	18.00293709	7.05236114	5.75917116
C	11.42137825	9.03174827	1.30769309
H	11.51704608	8.69778557	0.26604963
H	11.07680021	10.07471653	1.29596180
H	10.62408226	8.43288253	1.76922779
C	19.30285458	9.58547562	2.42886563
H	20.15424588	9.24603231	1.82097298
H	19.51133973	10.62395014	2.73078896
C	15.90703104	14.44101037	3.90564537
H	16.65449724	15.19155653	3.61200572
H	15.83004168	14.46661409	4.99969939
H	14.94369369	14.76326769	3.48823899
C	18.78299945	9.56608919	6.93772257
H	15.72294129	8.16297896	2.70305225

7. X-ray diffraction data for $[Fe(depe)_2]^+[BAr^F{}_4]^-$ ($[\mathbf{1}]^+[BAr^F{}_4]^-$).

The $[Fe(depe)_2]$ cation and the $[BAr^F{}_4]$ anion in the structure of $[\mathbf{1}]^+[BAr^F{}_4]^-$ were found to sit on independent –4 positions. With the exception of the Fe centre, the cation was found to be disordered due to a mismatch between the lower symmetry of the cation (D_2) and the site symmetry of its location in the crystal (S_4), hence only the Fe atom (which sits directly on the latter) is strictly ordered. This disorder was modelled by using one complete unique 50% occupancy orientation for the depe ligand. The geometry of the unique orientation was optimised, the thermal parameters of the phosphorus atoms were restrained to be similar, and all of the non-hydrogen atoms were refined anisotropically. Two of the CH_3 groups of each depe ligand orientate themselves above and below the FeP_4 moiety, which might suggest weak C–H···Fe agostic or anagostic interactions. While the inherent crystallographic disorder precludes a definitive assignment, it should be noted that computational calculations suggest the absence of such features.

Both of the unique CF_3 groups of the $[BAr^F{}_4]$ anion were found to be disordered. In each case two orientations were identified for the fluorine atoms, of *ca*. 91:9 and 87:13% occupancy for the C27- and C28-based groups respectively. The geometries of each pair of orientations were optimised, the thermal parameters of adjacent atoms were restrained to be similar, and only the atoms of the major occupancy orientations were refined anisotropically (those of the minor occupancy orientations were refined isotropically). Symmetry transformations used to generate equivalent atoms:

1: y+1/2, -x+3/2, -z+3/2

2: -y+3/2, x-1/2, -z+3/2

3: -x+2, -y+1, z

Crystal data for [Fe(depe)$_2$]$^+$[BAr$^F{}_4$]$^-$ ([**1**]$^+$[BAr$^F{}_4$]$^-$): [$C_{20}H_{48}FeP_4$]($C_{32}H_{12}BF_{24}$), *M* = 1331.54, tetragonal, *I*-4 (no. 82), *a* = *b* = 13.2989(3), *c* = 17.4417(6) Å, *V* = 3084.75(18) Å^3, *Z* = 2 (–4 symmetry), D_c = 1.434 g cm^{-3}, μ(Mo-Kα) = 0.454 mm^{-1}, *T* = 173 K, blue tabular plates, Agilent Xcalibur 3 E diffractometer; 3536 independent measured reflections (R_{int} = 0.0245), F^2 refinement,[11,12] R_1(obs) = 0.0466, wR_2(all) = 0.1067, 2993 independent observed absorption-corrected reflections [$|F_o| > 4\sigma(|F_o|)$, $2\theta_{max}$ = 58°], 266 parameters. The structure was refined as a two component inversion twin [Flack parameter *x* = +0.03(3)]. CCDC 1451414.

Formula	C52 H60 B F24 Fe P4	
Formula weight, *M*	1331.54	
Temperature	173 K	
Diffractometer, wavelength	OD Xcalibur 3, 0.71073 Å	
Crystal system, space group	Tetragonal, *I*-4 (no. 82)	
Unit cell dimensions	*a* = 13.2989(3) Å	α = 90°
	b = 13.2989(3) Å	β = 90°
	c = 17.4417(6) Å	γ = 90°
Volume, Z	3084.75(18) Å^3	
Z	2 (–4 symmetry)	
Density (calculated), D_c	1.434 g cm^{-3}	
Absorption coefficient	1.110 mm^{-1}	
F(000)	1358	
Crystal colour / morphology	Blue tabular plates	
Crystal size	0.44 x 0.22 x 0.07 mm^3	
θ range for data collection	3.064 to 29.134°	
Index ranges	-15<=h<=16, -17<=k<=17, -23<=l<=20	
Reflections collected / unique	11440 / 3536 [R(int) = 0.0245]	
Reflections observed [F>4σ(F)]	2993	
Absorption correction	Analytical	
Max. and min. transmission	0.970 and 0.896	
Refinement method	Full-matrix least-squares on F^2	
Data / restraints / parameters	3536 / 127 / 265	
Goodness-of-fit on F^2	1.039	
Final R indices [F>4σ(F)]	R1 = 0.0465, wR2 = 0.0990	
	R1+ = 0.0465, wR2+ = 0.0990	
	R1- = 0.0566, wR2- = 0.1232	
R indices (all data)	R1 = 0.0595, wR2 = 0.1060	
Absolute structure parameter	x+ = 0.029(8), x- = 0.971(8)	
Largest diff. peak, hole	0.294, -0.176 eÅ^{-3}	
Mean and maximum shift/error	0.000 and 0.002	

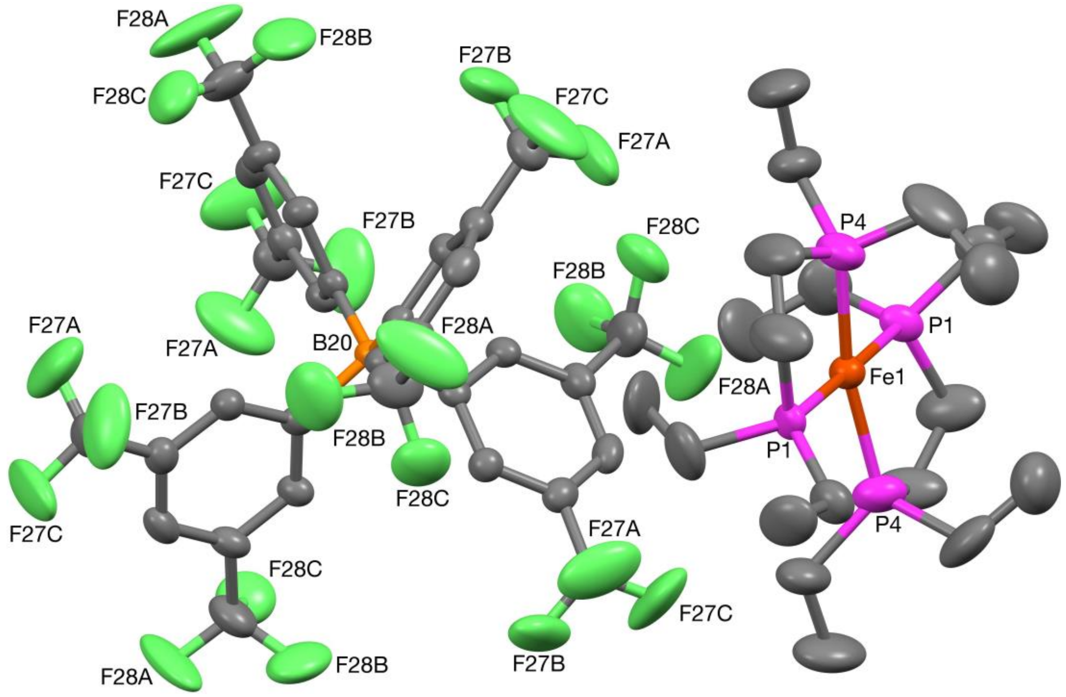

Figure S18. The crystal structure of [**1**]$^{+}$[BAr$^{F}{}_{4}$]$^{-}$ (50% probability ellipsoids). C atoms in grey. H atoms omitted for clarity.

8. References

[1] P. J. Hill, L. R. Doyle, A. D. Crawford, W. K. Myers and A. E. Ashley, *J. Am. Chem. Soc.*, 2016, **138**, 13521.

[2] A. Hills, D. L. Hughes, M. Jimenez-Tenorio, G. J. Leigh, A. T. Rowley, J. F. Sawyer, L. A. Oro, G. J. Kulas, P. J. Vergamini and K. G. Coulton, *J. Chem. Soc. Dalt. Trans.*, 1993, **24**, 3041.

[3] J. Chatt and R. G. Hayter, *J. Chem. Soc.*, 1961, 5507.

[4] I. Chávez, A. Alvarez-Carena, E. Molins, A. Roig, W. Maniukiewicz, A. Arancibia, V. Arancibia, H. Brand and J. M. Manríquez, *J. Organomet. Chem.*, 2000, **601**, 126.

[5] M. Brookhart, B. Grant and A. F. Volpe, *Organometallics*, 1992, **11**, 3920.

[6] D. F. Evans, *J. Chem. Soc.*, 1959, 2003.

[7] E. M. Schubert, *J. Chem. Educ.,* 1992, **69**, 62.

[8] R. Battino, T. R. Rettich and T. Tominaga, *J. Phys. Chem. Ref. Data*, 1984, **13**, 563.

[9] S. Stoll and A. Schweiger, *J. Magn. Reson.*, 2006, **178**, 42.

[10] G. M. Sheldrick, IUCr, H.-I. R., G. M., R. J., S. G. M., *Acta Crystallogr. Sect. A Found. Adv.*,

2015, **71**, 3.

[11] SHELXTL v5.1, Bruker AXS, Madison, WI, 1998.

[12] C. G. Balesdent, J. L. Crossland, D. T. Regan, C. T. López and D. R. Tyler, *Inorg. Chem.*, 2013, **52**, 14178.

[13] ADF2014, SCM, Theoretical Chemistry, Vrije Universiteit, Amsterdam, The Netherlands, http://www.scm.com.

[14] S. H. Vosko, L. Wilk and M. Nusair, *Can. J. Phys.*, 1980, **58**, 1200.

[15] A. D. Becke, *Phys. Rev. A, Gen. Phys.*, 1988, **38**, 3098.
